# Human interactions with tropical environments over the last 14,000 years at Iho Eleru, Nigeria

**DOI:** 10.1016/j.isci.2023.106153

**Published:** 2023-02-08

**Authors:** Jacopo Niccolò Cerasoni, Emily Yuko Hallett, Emuobosa Akpo Orijemie, Kseniia Ashastina, Mary Lucas, Lucy Farr, Alexa Höhn, Christopher A. Kiahtipes, James Blinkhorn, Patrick Roberts, Andrea Manica, Eleanor M.L. Scerri

**Affiliations:** 1 Department of Biology, Loyola University Chicago, Chicago, IL 60660, USA; 2 Pan-African Evolution Research Group, Max Planck Institute of Geoanthropology, 07745 Jena, Germany; 3 Department of Anthropology, Loyola University Chicago, Chicago, IL 60660, USA; 4 Department of Archaeology and Anthropology, University of Ibadan, 200132 Ibadan, Nigeria; 5 Department of Archaeology, Max Planck Institute of Geoanthropology, 07745 Jena, Germany; 6 Arctic University Museum of Norway, UiT-the Arctic University of Norway, 9019 Tromsø, Norway; 7 Department of Archaeology, University of Cambridge, Cambridge CB2 3DZ, UK; 8 Institute of Archaeological Sciences, Goethe-Universität, 60323 Frankfurt am Main, Germany; 9 Institute for the Advanced Study of Culture and the Environment, University of South Florida, Tampa, FL 33620, USA; 10 isoTROPIC Research Group, Max Planck Institute of Geoanthropology, 07745 Jena, Germany; 11 School of Social Science, the University of Queensland, Brisbane, QLD 4072, Australia; 12 Department of Zoology, University of Cambridge, Cambridge CB2 3EJ, UK; 13 Department of Classics and Archaeology, University of Malta, 2080 Msida, Malta; 14 Institute of Prehistoric Archaeology, University of Cologne, 50923 Cologne, Germany

**Keywords:** Biological sciences, Plant Biology, Paleobiology

## Abstract

The Ihò Eléérú (or Iho Eleru) rock shelter, located in Southwest Nigeria, is the only site from which Pleistocene-age hominin fossils have been recovered in western Africa. Excavations at Iho Eleru revealed regular human occupations ranging from the Later Stone Age (LSA) to the present day. Here, we present chronometric, archaeobotanical, and paleoenvironmental findings, which include the taxonomic, taphonomic, and isotopic analyses of what is the only Pleistocene faunal assemblage documented in western Africa. Our results indicate that the local landscape surrounding Iho Eleru, although situated within a regional open-canopy biome, was forested throughout the past human occupation of the site. At a regional scale, a shift from forest- to savanna-dominated ecotonal environment occurred during a mid-Holocene warm event 6,000 years ago, with a subsequent modern reforestation of the landscape. Locally, no environmental shift was observable, placing Iho Eleru in a persistent forested “island” during the period of occupation.

## Highlights

• Multi-proxy paleoecology of Pleistocene and Holocene human occupations at Iho Eleru
• Mid-Holocene warming led to regional shift from forest- to savanna-dominated ecotone
• Iho Eleru was located in a persistent forested “island” for the last 14,000 years
• Modern forested environment is likely a result of intensive anthropogenic agroforestry

## Graphical abstract

**Figure:**
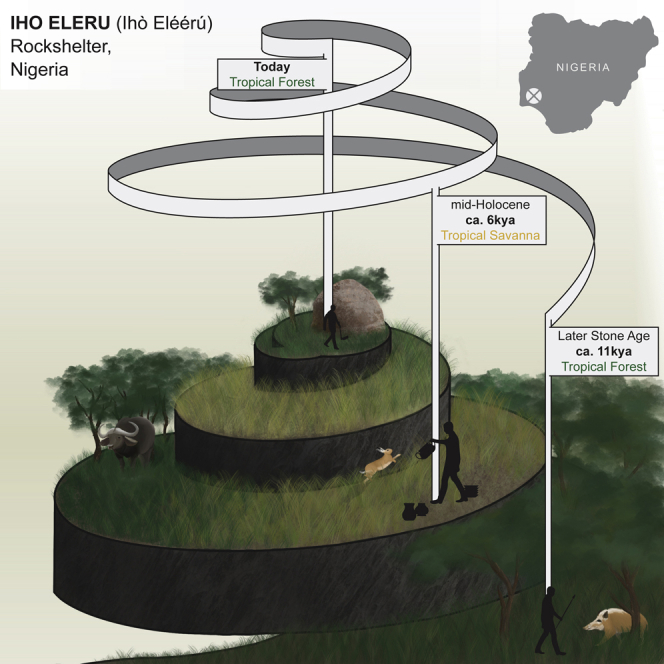

## Introduction

**Figure 1:**
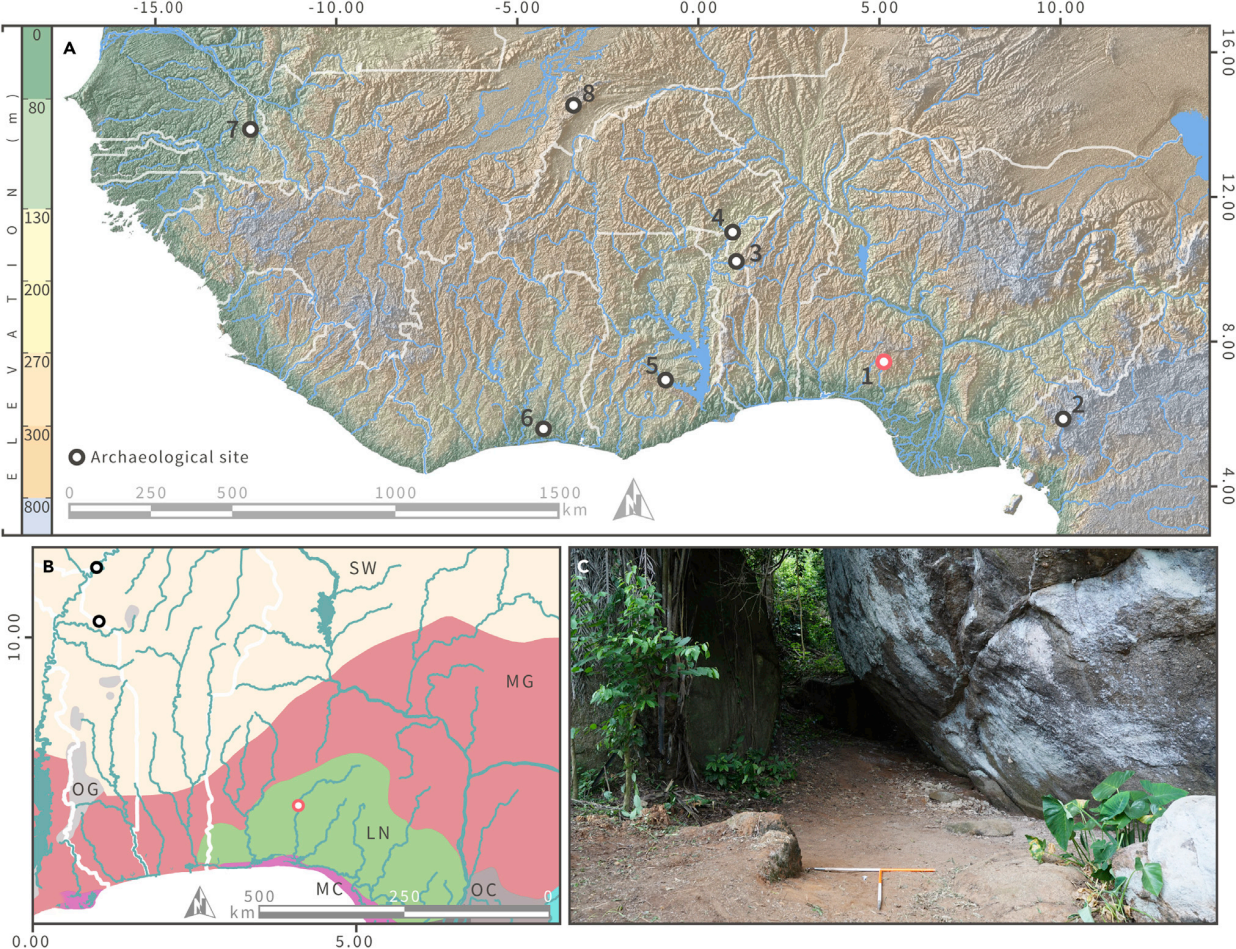
Geographical and ecological context of Iho Eleru (A) Map of western Africa showing river and lake systems (in blue), political boundaries (in white), geolocated position of Iho Eleru, and other LSA and early ceramic sites (1- Iho Eleru, 2- Shum Laka, 3- Koukou-I, 4- Pendjari-II, 5- Bosumpra Cave, 6- Bingerville Highway, 7- Toumboura II and Fatandi V, and 8- Ounjougou; Table S1, for further data see^9^), (B) Map of Iho Eleru and neighboring regions showing river and lake systems (in blue), political boundaries (in white), and modern ecoregions boundaries^9^ (SW- West Sudanian savanna, MG- Guinean forest-savanna mosaic, OG- Eastern Guinean forest, LN- Nigerian Lowland Forest, OC- Cross-Niger transition forests, and MC- Central African mangroves). (C) North-facing view of the plateau area of Iho Eleru rock shelter.

**Figure 2:**
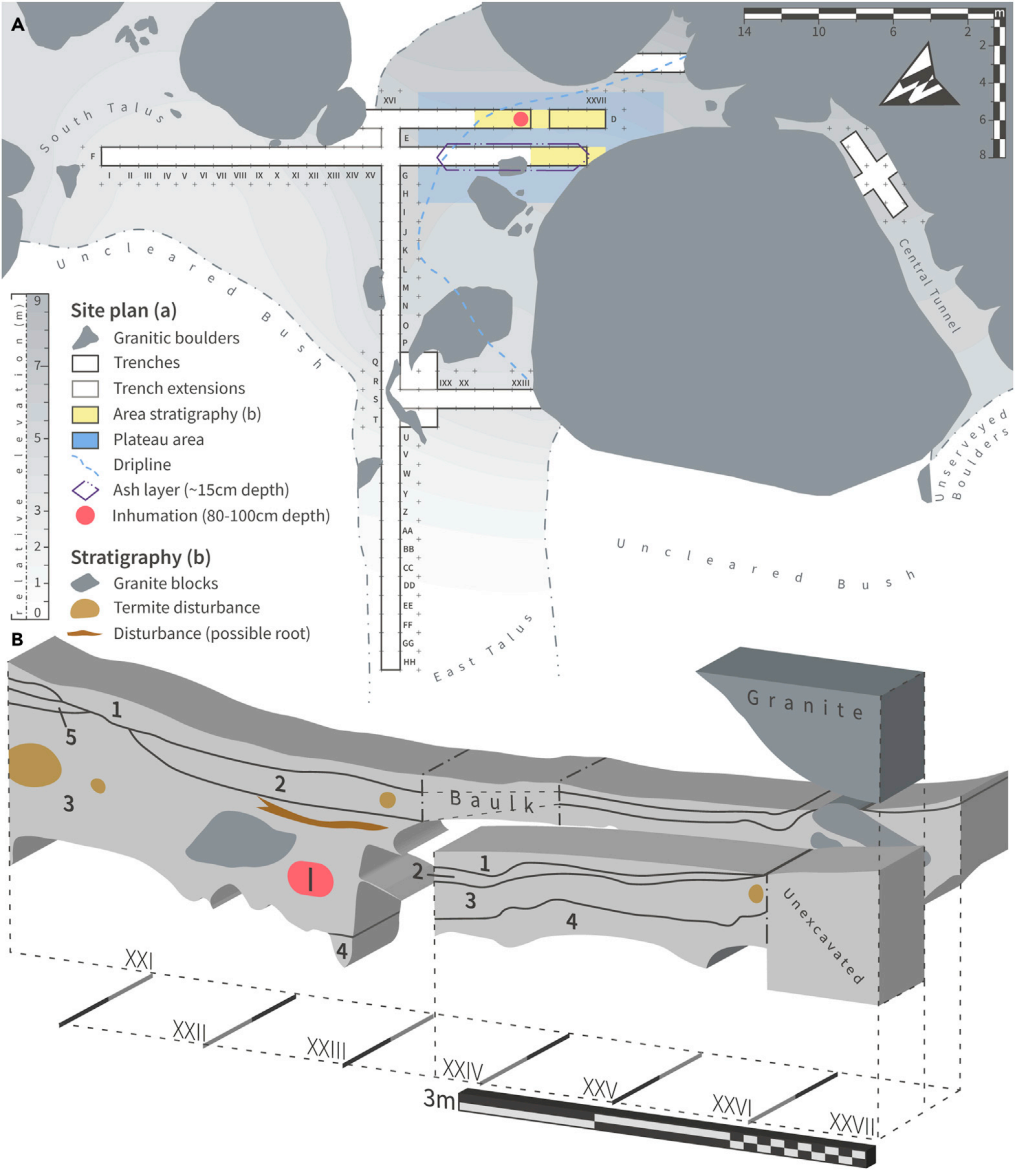
Site plan (A) of Iho Eleru and stratigraphic sections (B) of the upper plateau area (marked in yellow on the site plan), along the east faces of trench D XXI-XXVII, and trench F XXIV-XXVII The Pleistocene fossil inhumation is shown with a red circle on the plan (A) and with the letter “I” in the stratigraphic section (B). The stratigraphical layers were first described by Shaw and Daniels^1^ and are characterized by very slight changes in sediment color, appearance, and composition. Each layer was previously described as follows: 1 - layer composed of superficial (compacted) ashes, 2 - layer with compacted red sandy composition, 3 - layer with red-brown soil, 4 - layer with gravel, and 5 - layer with loose reddish-brown sandy composition (corrected and redrawn after Shaw and Daniels^1^; pp. 189 and 193).

The Ihò Eléérú (or Iho Eleru) rock shelter (7.441378, 5.124756: Figure 1) is a unique site located within an inselberg area near the northern provincial boundary of Ondo State, Nigeria, approximately 25 km north-west of the regional capital of Akure. First documented in the early 1960s by officers of the Nigerian Department of Antiquities, Iho Eleru was excavated by T. Shaw and S.G.H. Daniels^1^ between 1963 and 1964. The excavations revealed a c.1.8m deep stratigraphic sequence containing very high densities of cultural material, spanning from the terminal Pleistocene 13.2 thousand years ago (ka) up to today (Figure 2). Approximately half a million stone tools were recovered, together with a variety of faunal remains and the only Pleistocene hominin fossil ever discovered in western Africa.^1^ The latter has been extensively studied morphologically,^2^^,^^3^^,^^4^^,^^5^^,^^6^^,^^7^ and has been interpreted as a *Homo sapiens* individual with archaic features. Only one publication^6^ has documented the fossil’s stratigraphic and chronological context since the original excavation report.^1^ Furthermore, the stratigraphic provenance of the remaining material, although well recorded by the original authors, has not been utilized to place the findings within their regional paleoenvironmental and archaeological context.

Various localities in western Africa and neighboring regions report similar archaeological records, from the western-most areas of the Falémé Valley in Senegal to the high-altitude plateaus of Northern Cameroon^8^^,^^9^ (Figure 1). Most of these Later Stone Age (LSA; approximately 50,000 to 11,000 years ago) sites appear in open air contexts, such as Toumboura II, Fatandi V, Bingerville Highway, Koukou-I, Pendjari-II, and Ounjougou (Table S3). The only rock shelter sites which display LSA assemblages and have stratigraphies that cover the terminal Pleistocene-Holocene transitions are Iho Eleru, Bosumpra Cave, and Shum Laka. All of the sites, in both open air and rock shelter settings, are characterized by a variety of LSA “small tool” lithic types, such as microliths and foliates. Today, only the sites of Bingerville Highway, Shum Laka, and Iho Eleru are not located in savanna-dominated environments, which are characterized as a mixed woodland-grassland ecosystem.^10^ Furthermore, Bingerville Highway is the only site found within modern rainforests. Tropical rainforests, characterized by a majority of moist broadleaf trees,^10^ were widely inhabited during the LSA throughout Africa^11^^,^^12^^,^^13^ and sporadically during the Middle Stone Age (MSA, approximately 150,000 to 50,000 years ago) in Anyama, Ivory Coast.^14^ Uniquely, Iho Eleru is the only known site which is located near an ecotone between savanna and forest ecoregions (Figure 1). Considering these differences, it is clear that Iho Eleru is a highly significant site both in terms of archaeological characterization and environmental context.

In 2019, a re-evaluation of the original collection of excavated materials was conducted, including re-analysis of materials at the Department of Anthropology and Archaeology, University of Ibadan, Nigeria. Access and exporting of the original archaeobotanical and faunal collections were granted by the University of Ibadan, and the collections were exported to the Max Planck Institute for the Science of Human History, Germany, for further analysis.

## Results

In this study, we applied a multidisciplinary approach to better understand behavioral and environmental changes at Iho Eleru at a local and regional scale and the broader relevance of the site to discussions of Pleistocene and Holocene tropical forest habitat occupation and utilization of our species.^11^ The archaeobotanical collection was sub-sampled for chronometric and archaeobotanical analysis. The faunal collection, previously reported lost (Shaw and Daniels,^1^ p. 30), was analyzed by means of taphonomic, taxonomic, and isotopic techniques. Furthermore, paleoenvironmental reconstructions and paleoclimatic models were employed to gain a better understanding of regional environmental trends during the period of occupation. Similar multidisciplinary approaches to tropical caves and rock shelters have been applied successfully,^15^^,^^16^^,^^17^^,^^18^ improving our understanding of human-tropical environment interactions in the Pleistocene-Holocene time frame.

### Chronometric analysis

**Figure 3:**
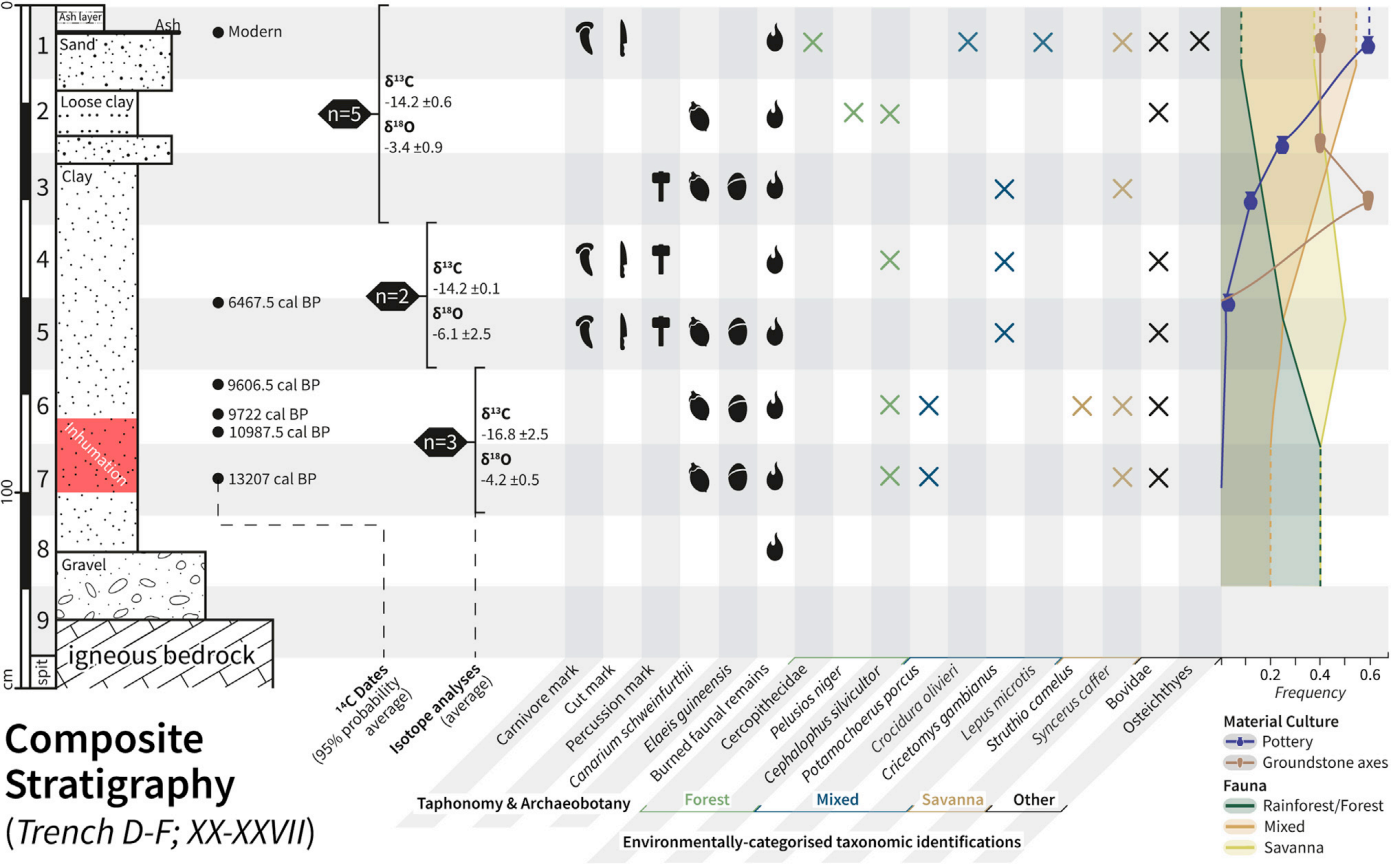
Composite stratigraphy of Trenches D and F, spits XX-XXVII Within it are presented ^14^C dates, the isotopic results averaged into three discrete groups, archaeobotanical identification of *C. schweinfurthii* and *E. guineensis*, taphonomic identification of the faunal assemblage, environmentally categorized taxonomic identifications, and frequency of major environmentally categorized taxonomic groups and material culture (pottery and ground stone axes).

The summarized ^14^C dating results for charred plant macroremains and faunal remains sampled from the plateau area of the rock shelter are shown in Figure 3.

We applied modern radiocarbon dating methodologies to the recovered archival charred plant material to verify the original results of the Shaw and Daniels radiocarbon dating program.^1^ The resulting dates (Figure S1 and Table S4) span a wide time frame, from c.13,200 cal. BP (Before Present) to recent. The dating results we obtained were remarkably close to the original dates offered by Shaw and Daniels, and, similarly, those complement previously reported U/Th age estimates obtained directly from the Iho Eleru hominin skeletal material.^7^ These results offer a reliable radiocarbon chronology for the sedimentary context of the remains. Owing to partial stratigraphic inversion, several of the dates for this study are in disagreement with the original dates. Some of the sedimentary contexts at the site, specifically located within the taluses and external plateau areas of the rock shelter (Figure 2), are interpreted as sediments with post-erosional origins. To accommodate such inversion and erosional activities, we only considered the stratigraphy and dates from Trenches D and F, squares XX-XXVII, as reliably *in situ*.

### Archaeobotany

Out of 21 analyzed archaeobotanical samples (Table S6), 12 contained identifiable wood charcoal fragments, six contained endocarp fragments of (cf.) *Canarium schweinfurthii*, eight contained (cf.) *Elaeis guineensis*, and 12 contained other unidentifiable fragmented carbonized plant remains. Direct dating on the endocarps demonstrates early exploitation of canarium and probable oil palm from before 10 ka, with the earliest exploitation of *Canarium schweinfurthii* in western Africa, directly dated to ∼11.3 cal. BP (sample IW2792, Table S4). Fourteen different charcoal types were distinguished among the 82 analyzable wood fragments (see STAR Methods for details on botanical nomenclature). Owing to preservation and fragment size, identification reached (probable) genus level in seven cases. Definite association of a charcoal type to forest trees is so far only possible for the sample dated to c. 400 years ago (STROMBOSIA spp./STROMBOSIOPSIS spp.) and ZANTHOXYLUM spp. from spit 3. The cf. identification of charred GUAREA spp. and PIPTADENIASTRUM AFRICANUM (spit 5 and 6) also supports the exploitation of forest vegetation with possible use of their wood and edible organs. Other samples include possible forest and/or woodland taxa. No charcoal type clearly representing a savanna taxon was identified.

Further archaeobotanical investigation was carried out, with the objective of identifying paleobotanical microfossils from loose sediments present in the collected archaeobotanical samples. Out of 21 archaeobotanical samples, 13 contained trace amounts of sediment, with a varying amount between 1 and 10 g each. These samples were processed for phytolith extraction and analysis. Unfortunately, no phytolith or other archaeobotanical microremains were identified based on the protocol used. The lack of microremains might reflect the taphonomic pathway of the sediment samples available to us, post-depositional degradation of these materials at the site, or degradation occurring post-excavation while the samples were in storage over the last half a century. Further investigation of *in situ* deposits will be required to confirm whether or not phytoliths can be successfully retrieved from the sediments at Iho Eleru.

### Fauna

**Figure 4:**
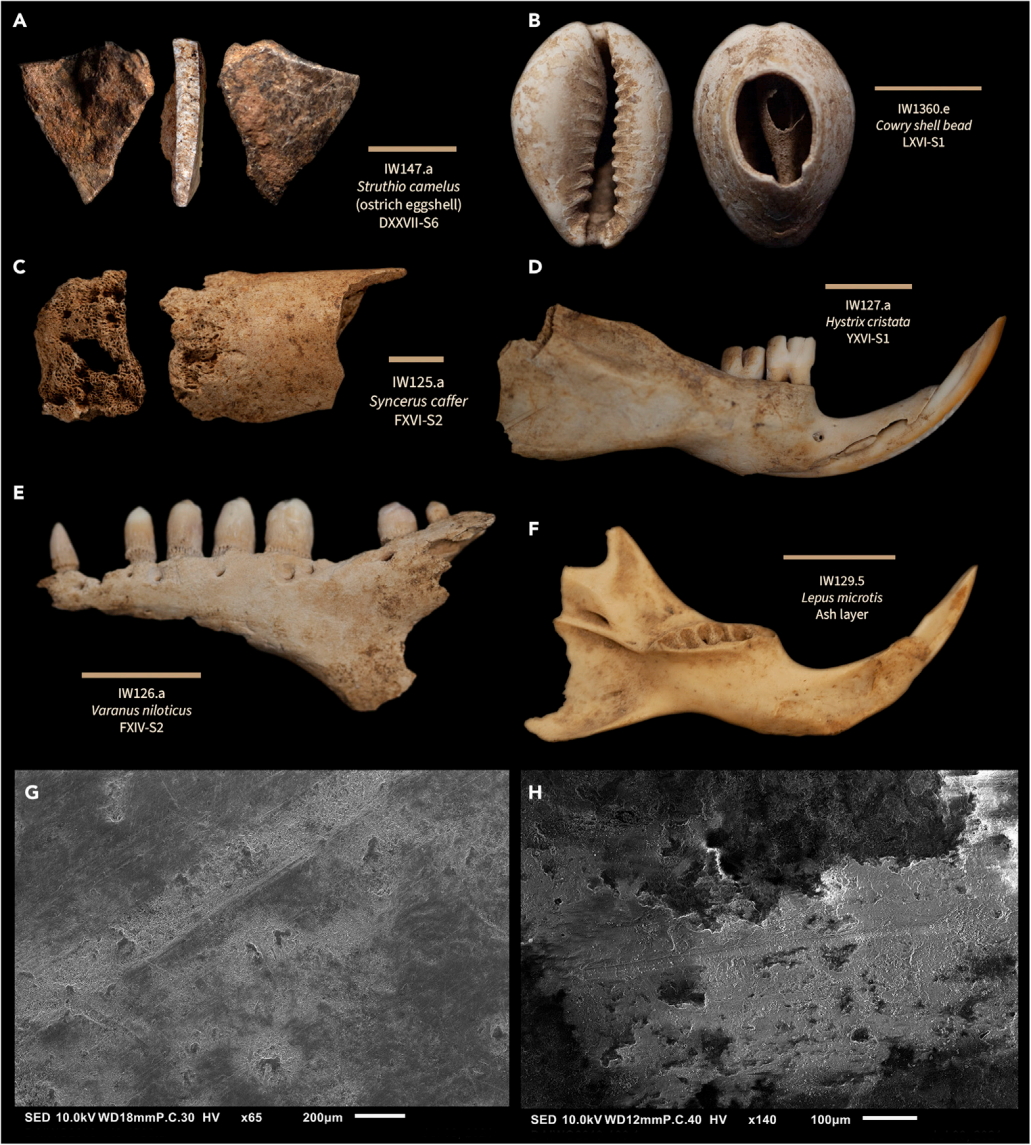
Selection of photographs of faunal remains and SEM of cut/percussion marks A selection of faunal remains (A–F) from taxa identifiable as either forest or savanna dwelling. One tooth sampled for isotope analysis is shown (F). Scales are 1 cm. SEM images of cut marks are presented on specimens from layers DXXVI-S1 (G) and FXXI-S5 (H).

The formation processes of the Iho Eleru vertebrate faunal assemblage were analyzed through taphonomic investigation of all bone and tooth fragments. Our goal was to determine the extent of human-accumulated bone relative to carnivore-accumulated bone and to reconstruct the foraging and butchery behavior of humans over time. Of a total 207 retrieved bone fragments originating from the whole site, only 152 had intact bone surfaces that could be analyzed for bone surface modification, burning, and breakage patterns (Table S7). The Iho Eleru faunal assemblage is highly fragmented, with the average analyzed fragment length measuring 2.67 cm, width at 1.36 cm, and thickness at 0.88 cm. Burned bone fragments constitute 28.9% (N = 44) of the assemblage, and the majority of these are carbonized, while few are calcined. Despite the small sample size of the assemblage, butchery marks in the form of percussion marks, percussion notches, and cut marks were present on 12.5% (N = 19) of the bone fragments. The rate of carnivore accumulation was similar, with 11.8% (N = 18) of the bone fragments bearing tooth marks or tooth notches and 3.29% (N = 5) displaying gastric etching from carnivore digestion. These rates of surface modification preservation are low in comparison to other Late Pleistocene African archaeological^19^^,^^20^ (also see^21^ for comparable rates of surface modification), experimental, and actualistic assemblages (Marean and Kim,^22^) and are likely the result of significant post-depositional fragmentation^23^ and poor preservation (i.e., acidity and soil moisture content) in a tropical context. Anthropogenic butchery marks and carnivore tooth marks were identified on *Syncerus caffer* (African buffalo), *Lepus microtis* (African savanna hare), *Hystrix cristata* (crested porcupine), and other taxonomically unidentifiable remains (Figure 4). Surface modification identification was completed for each bone fragment, and the results are presented in Table S7. No worked bone was identified.

Taxonomic identification of the Iho Eleru vertebrate faunal assemblage documented ten taxa to the species level. The identified species prefer habitats ranging from forests to savannas and reveal the ecotonal nature of the exploited area surrounding the rock shelter. A complete list of identified taxa is shown in Table S8. The Iho Eleru vertebrate faunal assemblage includes taxa ranging in body size from the large *S. caffer* (African buffalo) to the small *Pelusios niger* (West African black turtle). Identified vertebrates that today prefer forest habitats include *Cephalophus silvicultor*^24^ (yellow-backed duiker), *Potamochoerus porcus*^24^^,^^25^ (bushpig), *H. cristata*^24^ (crested porcupine), *Cricetomys* sp.^24^ (giant pouched rat), and *Varanus niloticus*^26^ (Nile monitor). Based on the number of identified specimens (NISP), the relative frequency of forest vertebrates recovered from the Iho Eleru assemblage decreased over time (Figure 3), while the relative frequency of savanna vertebrates increased over time (Figure 3). Identified vertebrates preferring savanna habitats include *S. caffer*^27^ (African buffalo), *L. microtis*^24^ (African savanna hare), and *Struthio camelus*^28^ (ostrich). A single burned *S. camelus* (ostrich) eggshell fragment was identified in D27 spit 6, suggesting that either savanna was in close proximity to the site, as ostrich strictly occupy open grassland habitats, or that ostrich eggshell was transported to the site to be consumed by humans or to be used as a water canteen^29^ or an ornamental object.^30^ No carnivore remains or coprolites were identified, although carnivores did apparently act as agents of bone accumulation. A total of six Osteichthyes (bony fish) bone fragments and two marine shells were identified in modern deposits only, and no terrestrial gastropods were identified as they were originally isolated from the excavated material for independent analysis shortly after the original excavation.^1^

**Figure 5:**
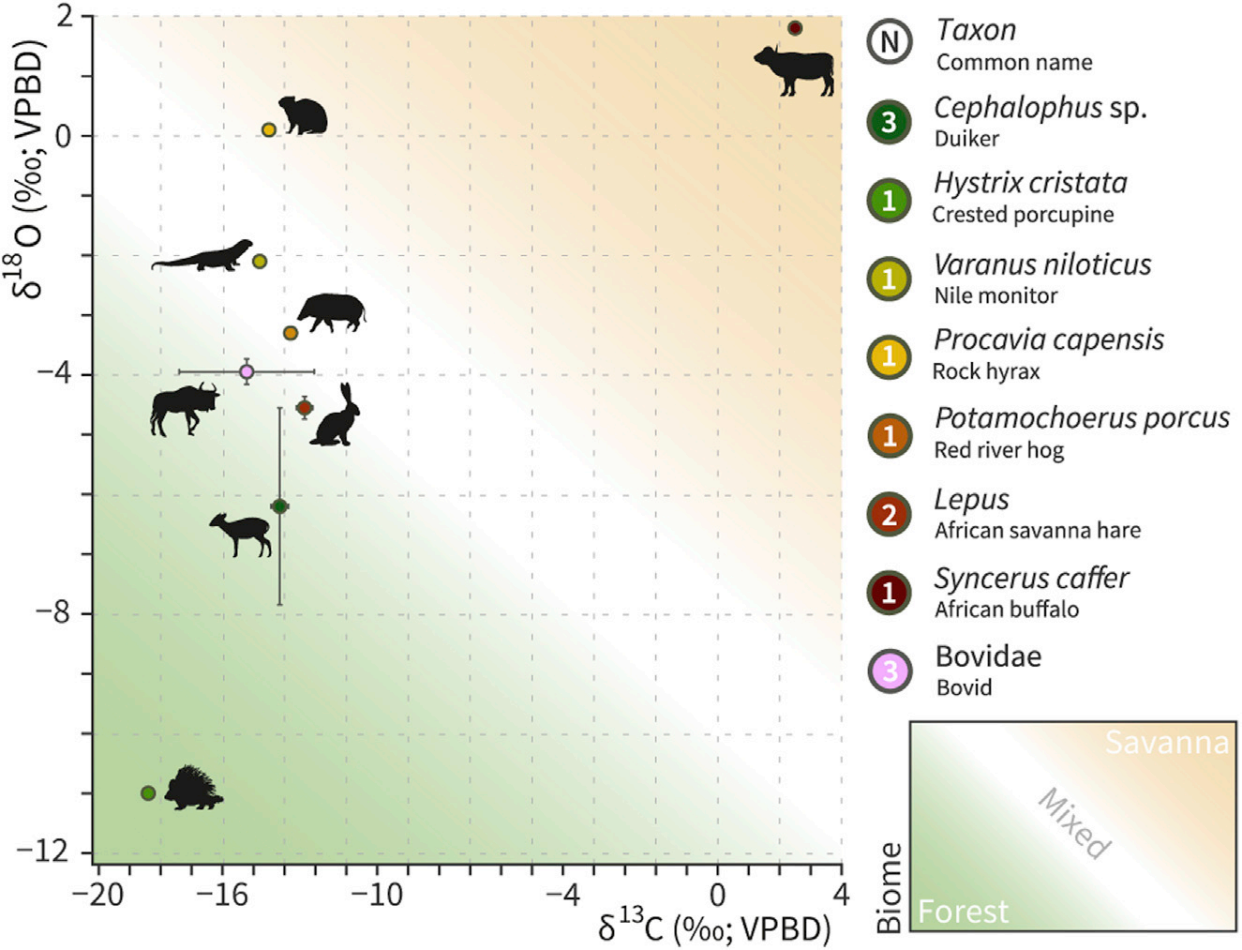
Stable carbon and oxygen isotope data for all faunal samples analyzed (n = 13) Results table visible in Table S4. Mixed feeders both graze in grassland and browse in shrubland and forests.^31^

The δ^13^C and δ^18^O stable isotope results for all 13 analyzed vertebrate teeth enamel samples from the site of Iho Eleru are shown in Figure 5 and Table S5, with photographed examples in Figure S3. Although the sample size is limited, primarily by the number of teeth present in the assemblage, the δ^13^C values cover a wide range (−18.6‰–2.5‰), suggesting that these animals exploited a variety of habitats, from closed-canopy C_3_ forests to open C_4_-dominated grasslands. When we compare our isotopic data to documented modern habitat preferences for the sampled vertebrate species, the δ^13^C values are consistent with ecological expectations, with *S. caffer* plotting as an outlier and signaling a preference for savanna (Figure 5). The δ^18^O values covered a similarly wide range (−11.0‰–1.8‰), suggesting a variety of groundwater and food water sources for the animals present, consistent with access to a variety of environments ranging from shaded forests to more open, arid settings. Stratigraphically, both δ^13^C and δ^18^O values remain largely consistent throughout the stratigraphy, with elements of closed-canopy forest and ecotonal forest-savanna habitats (e.g., *P. porcus*, *H. cristata*, and *L. microtis*), and some sporadic elements of grassland habitat (e.g., *S. caffer*) exploited by animals or procured by humans living at the site. Overall, the stable isotope results from vertebrate teeth sampled from Iho Eleru reveal greater proportions of forest habitats present at the site. Although the small sample size and taphonomic bias may contribute to this trend, this may be indicative of the human habitation of a forest island within a more mosaic habitat landscape.

## Discussion

Throughout the terminal Pleistocene and Holocene, western Africa was a culturally variable region, a region that was home to a variety of cultural-environmental processes, with synchronous presence of MSA,^32^ LSA, and ceramic cultures in different parts of the region. Our results show that Iho Eleru, characterized by LSA and some of the earliest West African ceramic culture, underwent regular occupation during the documented timeline, with focused use of resources from both forest and grassland environments. The archaeobotanical and isotopic data suggest a closed-canopy environment in the close proximity of the site, as evidenced by the lack of retrieved savanna taxa charcoal remains (Table S6) and the average isotopic results (Figure 3). Although we must be mindful that different proxies can be biased as a consequence of human selection and use of the landscape, particularly in terms of the inhabited range of some faunal species. Similar studies^33^^,^^34^ carried out on equatorial latitudes of western Africa show a correlation between inselberg areas, such as that where Iho Eleru is located, and forested environmental footprints within them compared to their surrounding environment. Indeed, it is clear that humans valued forest resources from their first arrival at the site given the supporting evidence to their exploitation of forest-dwelling species. Nevertheless, our data also show a divergence in ecological and behavioral trends in regional and local scales.

The regional area around Iho Eleru seems to have persisted as a mixed tropical forest-savanna ecotonal landscape from the Late Pleistocene to the Late Holocene, where vegetation compositions varied in their proportion of forest and savanna, with the latter expanding at the onset of the warm mid Holocene (ca. 6 ka) as a result of changes in regional precipitation. The increase in identified non-arboreal pollen (NAP)-producing species in a multitude of regional cores around 5-6 ka suggests a similar increase in savanna-dominated environments across the region^35^^,^^36^^,^^37^^,^^38^ (Figures S5–S8). The regional and local proxies compared in this study indicate three major environmental phases that occurred during the timeline of recorded human activity at Iho Eleru. The first, the terminal Pleistocene before 12 ka, was characterized by a wetter environment than today, with increased levels of precipitation and humidity. This aligns well with the African Humid Period^39^^,^^40^^,^^41^ dated to 14.8–5.5 ka. The second, which occurred in the Middle Holocene, was characterized by an increase in temperature and a decrease in precipitation, as supported by a local increase in savanna-dwelling taxa and our modeled environmental and climatic proxies. This event occurred approximately 6 ka and can be interpreted as having caused an expansion of open-canopy environments.

Finally, the third event, which encompasses the present, is a yet-unexplained phenomenon characterized by the contrasting results our proxies show in this study. The high concentration of savanna-dwelling faunal species in modern layers and paleoclimate models suggest that the surrounding environment of the modern landscape should be a mixed environment with open- and closed-canopy environments (Figures S4 and S5). Nevertheless, modern day observations of the local landscape (within 1-5 km range) around Iho Eleru show a densely forested environment.^10^^,^^42^ This incongruence could be explained by one of two hypotheses: (1) just as observed in the paleoenvironmental record, the modern landscape is composed by a dominant open-canopy landscape with micro-scale forested islands, within which Iho Eleru is situated, or (2) the modern local landscape is not a product of its environmental and climatic conditions but is undergoing landscape modification through agroforestry practices by local populations (e.g., decreased fire regime, presence of grazing animals, and planting of useful tree species such as *Theobroma cacao* or cocoa tree, *Musa* spp. or plantain, and *E. guineensis* or palm oil). Comparable case studies present published evidence of anthropogenic modification of landscapes at similar degrees both in tropical Africa,^41^ South America,^43^^,^^44^ and Australia.^18^

In terms of human behavioral trends, the observed 6 ka warm mid-Holocene period may have influenced human behavior at the site. At ca. 6 ka a change in material culture is visible with the appearance of relief motif pottery and ground stone axes^1^ (Figure 3). This trend, also in concordance with the faunal and regional environmental information, suggests the arrival at Iho Eleru of new populations or material traditions, possibly aided by the increased presence of savannas and the relative contraction of forests. Our data also suggest that this subtle environmental shift was most likely not caused by anthropogenic processes (see^18^^,^^43^^,^^44^ for examples of anthropogenic processes), such as forest clearing from the use of controlled fires or ground stone axes, but rather by a change in precipitation rates and temperature as shown by the paleoenvironmental reconstruction and paleoclimatic models. Furthermore, the appearance of comparable material cultures is also visible in other sites within close proximities. Similar to Iho Eleru, a shift in material culture has been identified at Shum Laka, Cameroon, between 6 and 7 ka,^45^ where a “less humid” period was identified,^46^ and in which pottery and *Canarium schweinfurthii* first appeared. Other Holocene and LSA sites in western Africa with early ceramic traditions suggest an influx from the western part of the region, with the earliest appearance of pottery around 11.9 ka in Ounjougou, Mali,^47^^,^^48^ and a slightly later appearance at Bosumpra, Ghana^49^ (Figure 1).

Despite these regional and behavioral changes, it is clear that both tropical forest and savanna environments remained important for humans at Iho Eleru, particularly for the available water sources and starchy plants^50^^,^^51^ in forests and protein- and fat-rich fauna resources in savannas.^52^^,^^53^ The uniqueness of this site, both in terms of the exceptional retrieval of bioarchaeological and material cultural finds and because of its location in tropical western Africa, offers a new look on past human activity and behavior in tropical African environments. Nevertheless, further studies will be necessary to fully explain the dynamics of these environmental processes which affected, and were affected by, past and modern people at Iho Eleru.

### Limitations of the study

In the current study, it was not possible to evaluate the stratigraphical sedimentation of the site *in situ*. In addition, the preservation of the archaeological assemblage during storage caused deterioration of some samples, making them unsuitable for archaeobotanical, faunal, and isotopic analysis.

## STAR★Methods

### Key resources table

**Table:** 

REAGENT or RESOURCE	SOURCE	IDENTIFIER
**Deposited data**		

Archaeofaunal remains from Iho Eleru, raw and analyzed data	This paper	Tables S7 and S8
Palaeobotanical remains from Iho Eleru, raw and analyzed data	This paper	Tables S4, S6, and Figure S2
Chronometric data from Iho Eleru, analyzed data	This paper	Figure S1 and Table S4
Isotopic data from Iho Eleru, raw and analyzed data	This paper	Table S5 and Figure S3
Paleoclimatic reconstruction modeling	This paper	Figures S4, S5, and Data S1
Regional Palynological Record Modeling	This paper	Figures S5–S8 and Data S2

**Software and algorithms**		

R Studio	R Studio Team	https://www.rstudio.com/
R	R Core Team	https://www.r-project.org/
OxCal	University of Oxford	https://c14.arch.ox.ac.uk/oxcal.html
Illustrator 2022	Adobe Inc.	https://www.adobe.com/products/illustrator.html
Photoshop 2022	Adobe Inc.	https://www.adobe.com/products/photoshop.html

### Resource availability

#### Lead contact

Further information and requests for resources should be directed to and will be fulfilled by the lead contact, Jacopo Niccolò Cerasoni (jcerasoni@luc.edu).

#### Materials availability

The faunal and archaeological materials from Iho Eleru that were analyzed in this study are currently curated at the Max Planck Institute of Geoanthropology, Jena (DE). The paleobotanical materials from Iho Eleru that were analyzed in this study are currently curated for further analysis at Goethe-Universitat, Frankfurt (DE).

### Experimental model and subject details

All necessary permits for archaeological excavation and analysis were obtained from the Nigerian National Commission For Museums and Monuments (Number RL113109976NG). All archaeological samples were curated in Ibadan, Nigeria, in sterile plastic bags and given unique specimen identifiers.

### Method details

#### Radiocarbon dating (Table S4 and Figure S1)

Sampling and dating were carried out by the Curt-Engelhorn-Center Archaeometry gGmbH. For the bone samples collagen was extracted (modified Longin method), purified by ultrafiltration (fraction >30kD) and freeze-dried. For the plant macroremains pre-treatment using ABA-Method (Acid/Base/Acid, HCl/NaOH/HCl) was used. The insoluble fraction was used for further treatment. Following pre-treatment, the sample material was combusted to CO2 in an Elemental Analyzer (EA). CO2 was then converted catalytically to graphite. ^14^C were analyzed using a MICADAS-type AMS system in-house. The isotopic ratios ^14^C/^12^C and ^13^C/^12^C of samples, calibration standard (Oxalic Acid-II), blanks and control standards were measured simultaneously in the AMS. ^14^C-ages were ormalized to ^13^C = −25‰ and calibrated using the dataset IntCal20 and software SwissCal (L. Wacker, ETH-Zürich). Calibration graphs are generated using the software OxCal (small deviations between SwissCal and OxCal results are possible) (Table S4).

#### Archaeobotanical analysis (Table S6)

The archaeobotanical samples were excavated from Shaw and Daniels^1^ from soil sieved with a mesh-size of 0.635 cm (0.25 inches) and mostly labeled (wood) charcoal sample. The samples contain fragments of wood charcoal and/or other arbonized botanical macro-remains. Many wood charcoal fragments show breaks that have occurred during sieving and storing and are too small for anthracological analyses. Wherever possible wood charcoal was analyzed using a reflected light microscope (Leica DM4000M) at different magnifications, 50× – 500x, in dark and bright field, after manually fracturing the charcoal fragments along the three planes: transverse, longitudinal tangential and longitudinal radial. The identification process follows the steps described in Höhn & Neumann^54^ and relies on the Inside Wood database,^55^ the wood reference collection of the Goethe University Frankfurt (JWGw), and wood anatomical atlases.^56^^,^^57^^,^^58^ The names of the charcoal types are given in small capitals to clearly discriminate the anatomical charcoal types from the botanical taxa with which they are not necessarily identical.^54^^,^^59^^,^^60^ Most type descriptions are published and cf. PIPTADENIASTRUM AFRICANUM resembles PARKIA spp. But has some septate fibers in addition,^54^^,^^60^^,^^61^ ZANTHOXYLUM spp. Is documented in Figure S2. Carpological macro-remains were identified with a dissecting microscope (Leica S6D). Identification was achieved by comparison with specimens in the modern reference collection of the Laboratory of African Archaeobotany at Goethe University, Frankfurt am Main and with carbonized carpological material from Dibamba, Cameroon, identified by Stefanie Kahlheber (unpublished).

#### Phytolith analysis method

The archaeobotanical samples containing sediment within their original storage bags were processed for sediment isolation using sieve meshes, with varying grid sizes between 1 and 0.25 cm. For the phytolith extraction, the samples were first deflocculated using a sodium hexametaphosphate and warm water solution. For every 10 mL of soil, 100 mL of solution was used (ratio 2:90, sodium hexametaphosphate:water). Any sample which did not correctly deflocculate after 72 h within the solution was manually crushed with a mortar and pestle. Clays were then removed from the samples by gravity sedimentation, with a 1:10 ratio of solution to water, letting it stand for 1 h and then removing supernatant liquids. The clay removal process was repeated 3 times. The resulting clay-free samples were then fractioned by wet sieving and dividing the samples into sands (mesh sieve with grid size of 250 μm) and silts (mesh sieve with grid size of 50 μm). A centrifuge (1500 rpm for 10 min) and distilled water were then used to repeatedly wash the silt samples. The sand samples were put aside and not used for the rest of the process. Given the high contraction of carbonates in the higher spits of the stratigraphy, HCl was used to remove any carbonate particles. This was done by carefully adding HCl to the samples until no reactions were visible. The samples were then rinsed three times with distilled water and a centrifuge at 1700 rpm for 10 min each. Organics were then removed using a 30% H2O2 solution and placing the samples within the solution in a hot water bath at 40°C. Given the low percentage of organic materials in the samples this step was concluded after 2 h in the hot water bath, with the second hour reaching a temperature of 80°C. The samples were then rinsed five times with distilled water and a centrifuge at 1700 rpm for 10 min each. The resulting samples were then processed for any phytolith extraction by flotation using Sodium Polytungstate (SPT-1). A heavy liquid was created using a hygrometer at a specific density of 2.3 g/mL. The SPT-1 solution was then mixed with the samples and centrifuged at 1700 rpm for 5 min. The materials which were floating at the top were then removed and then rinsed three times with distilled water and a centrifuge at 1700 rpm for 10 min each. Next, the samples were dried using acetone, which was mixed with the samples (approximately 10 mL for 1 mL of soil), stirred and centrifuged at 1500 rpm for 5 min. Supernatant acetone solution was removed, and the process repeated twice more. The final samples were then left to dry in a fume cupboard for 2 nights. Finally, the resulting materials were mounted on slides by mixing silicon oil with the samples, placing a few drops of the solution on each slide, and then covering with the slide cover and using clear nail polish to seal them. The slides were analyzed with a Olympus BX53M microscope at 40–100× magnification.

#### Taphonomic and taxonomic identification of vertebrate faunal assemblage (Tables S7 and S8)

Shaw and Daniels^1^ report using ¼ inch sieves for excavated material, and sorting all vertebrate bone fragments according to context. To the best of our knowledge, all sieved material was preserved and no unidentifiable bone fragments were removed from the curated materials. The bone fragments were not cleaned in the present study, as no loose sediment adhered to bone surfaces. The initial phase of taphonomic recording was completed using an Olympus 10–40X zoom binocular microscope with high incident light. For final verification of cut marked and percussion marked bones, SEM (Scanning Electron Microscope) imaging was completed using a JEOL InTouch Scope JSM-IT100LA compact SEM Each bone fragment was analyzed for surface modification. This microscopic method of surface modification recording has shown 95% accuracy in blind tests,^62^ and allows zooarchaeologists to distinguish between cut marks, percussion marks, and tooth marks. Trampling and biochemical marks,^63^^,^^64^ such as chemical, insect, and root etching,^65^^,^^66^^,^^67^^,^^68^ were also recorded. Burning severity was recorded using bone surface colors that indicate burning in either a reducing or oxidizing atmosphere as: no burning, light (dark brown to black color), medium (black to dark gray color), heavy (gray color with shallow cracks), or calcined (white color with deep cracks). Burning extent was recorded as the proportion of a bone fragment displaying signs of burning. For each bone and tooth fragment that was not covered in an adhering carbonate matrix, the following variables were also recorded (see Table S7): measurements (length, width, thickness, or height for tooth crown height), skeletal element, element portion, side (left of right), taxonomic identification to the lowest level, circumference of fragment,^66^ surface visibility, exfoliation of bone surface, dendritic etching,^65^^,^^68^ pocking, sheen, smoothing, cut marks, percussion marks, percussion notches, tooth marks, tooth notches, rodent gnawing,^65^ weathering,^69^ fracture outline and angle,^70^ presence of fresh break(s) from excavation or post-excavation,^70^ and color. Taxonomic identifications of the vertebrate remains were completed using measurements and photographs collected in 2013 from modern reference collections stored at the American Museum of Natural History in New York. Published reference material^71^^,^^72^^,^^73^^,^^74^^,^^75^^,^^76^ was also used extensively to aid in vertebrate species identifications.

#### Scanning electron microscopy

SEM creates a high-resolution image of a surface by scanning it with a focused beam of electrons. In order to closely evaluate characteristics of the cut marks and other surface features, morphological analysis of the specimens was carried out in a scanning electron microscope (SEM) Jeol JSM-IT100 at the Microscopy and Paleobotany Laboratory of the Department of Archaeology at the Max Planck Institute of Geoanthropology, Jena.

Samples were fixed on a 32 mm plain specimen holder with a conductive one-sided copper adhesive tape. For artifacts larger than 30 mm we used a specimen holder with a plain station; artifacts smaller than 30 mm were mounted on either aluminum cylinders or aluminum pin stubs. All samples were imaged uncoated. The SEM was operated in a high vacuum mode at an accelerating voltage of 10 kV. Specimens were visualised using an in-lens secondary electron detector (SED) at magnifications from 22× to 250x. Depending on the size of the artifact, we attuned working distance (WD) from 11 to 18 and probe current (P.C.) from 30 to 44. Brightness, contrast, stigma, and focus were adjusted manually for each sample in the InTouchScope program.

#### Stable isotope analysis

Enamel sampling for δ^13^C and δ^18^O analysis was conducted at the Max Planck Institute of Geoanthropology (MPI-GEA). Tooth samples were first cleaned by air abrasion to remove surface contaminants. Roughly 8 mg of enamel powder was then drilled from the buccal surface of the tooth to gain a sample representing the full growth period of the tooth. The drilled enamel powder was then pre-treated with a 1% NaOH solution for 1 h. Samples were then rinsed, vortexed, and centrifuged three times with MilliQ water. 0.1M acetic acid was then added to the samples for 10 min followed by the rinsing process with MilliQ water. After pre-treatment, samples were frozen overnight before being freeze dried for 4 h. Samples were analyzed on a Thermo Gas Bench 2 connected to a Thermo Delta V Advantage Mass Spectrometer. Roughly 3 mg of each sample was weighed into borosilicate glass vials. The vials were then flushed with helium at 100 mL/min for 10-min 20ul of 100% phosphoric acid was then added to each sample and left to react for 1 h. Samples were calibrated using a three-point calibration with international standards IAEA NBS 18: δ^13^C −5.04‰, δ^18^O −23.2‰, IAEA 603: δ^13^C + 2.46‰, δ^18^O −2.37‰, and IAEA CO8: δ^13^C −5.764‰, δ^18^O −22.7‰. Replicate precision of standards was used to measure machine error where δ^13^C ± 0.2‰ and δ^18^O ± 0.2‰. Overall measurement precision was studied through the measurement of repeat extracts from a bovid tooth enamel standard (n = 20, ±0.2‰ for δ^13^C and ± 0.4‰ for δ^18^O). Generally, tropical environments are expected to produce relatively low δ^18^O values due to the high intensity of rainfall^77^ although values are heavily dependent on rainfall source.

#### Palaeoclimate reconstruction (Data S1)

We used the paleoclimate reconstructions based on the Hadley CM3 published in Beyer et al.24 to model changes in climate for the area surrounding Iho Eleru. We used the decimal coordinate location of Iho Eleru to extract climate variable values at 1.5° resolution from the Beyer et al.24 dataset in 1000-year intervals over the last 22,000 years (see Data S1). All climate variable values were then scaled together, and four climate variable values of interest were plotted in figure 6. These include BIO 1 (annual mean temperature), BIO4 (temperature seasonality), BIO 12 (annual precipitation), and BIO 15 (precipitation seasonality). Annual mean temperature and annual precipitation (BIO 1 and 12) were selected to have an average annual representation of the local climate and its effect on the local environment as precipitation and temperature are two main factors for vegetation growth and resilience. Temperature and precipitation seasonality were selected due to their effects on human resilience and agro-pastoral activities. Increased temperature and precipitation seasonality has a negative effect on agro-pastoral communities, as crop cultivation and livestock husbandry can be negatively impacted by drastic increases or decreases in temperature and precipitation.

#### Synthesised regional palynological (NAP) record modeling (Data S2)

To provide additional context to the faunal, anthracological, and macrobotanical remains at Iho Eleru, multiple pollen records from Nigeria, Benin, and the Atlantic Ocean were summarized into indices tracking regional trends in vegetation cover. Pollen records were acquired from an archived version of the African Pollen Database64, harmonised, standardised, and plotted in the R statistical computing environment (R Core Team, 2021). The code, data, and a detailed R markdown document are provided in supplementary Data 4. Using a custom function, the datasets are harmonized with a master reference. This includes updating old taxonomic nomenclature as well as addressing spelling errors. Anne-Marie Lézine currently maintains the master list, which is a product of the African Pollen Database.^40^ Data harmonisation includes associating pollen taxa with known PFTs (Plant Functional Types). Plant functional types can be used to group pollen taxa by growth form (arboreal trees/shrubs, herbaceous vegetation, lianas, arboreal palms, etc.) and make comparisons of records from different regions. Pollen records were harmonized and percentages of major plant functional types were calculated based on the total number of pollen identified (Figure S6). Our goal is to assess the relative proportion of forest and herbaceous vegetation cover (grasslands and savanna) and arboreal trees/shrubs and herbaceous PFTs are being used as proxies for these broad types of vegetation cover. Similar methods have been applied to regional assessments of vegetation change both for the African continent. After summarizing these records by PFT and calculating the percent of each major group, we compare the datasets by setting them on an equal scale using Z-scores, which represent the results as the difference from the mean in SD units. By doing so, we can compare fluctuations with small and large amplitudes on a uniform scale (Figures S3 and S4). The APD entry for Lac Sélé did not contain a radiocarbon chronology, so this code will either compute one using the Rbacon package or use an existing one. In order to create a single index to compare with the finds at Iho Eleru, we aggregated the Z-scores of arboreal and herbaceous PFTs from all of the records and binned them by chronological intervals. In Figures S7 and S8, the sample density for chronological bins representing 500 and 100 years are compared. This shows that, using 500-year bins, the minimum number of samples in a bin (at 20,000-19500 years BP) is two. The maximum is 14 and all of the Holocene bins (11,000 years BP to present) are represented by a minimum of six data points. amples were binned to 500-year intervals and the chronology was limited to the last 12,000 years. In order to evaluate whether our smoothing results were reasonable, multiple smoothing methods were applied to summarized results from both herbaceous and arboreal PFTs. All of the smoothing functions were calculated with 24 knots. Spline smoothing was calculated using R’s smooth.spline function. Kernel-density smoothing was calculated using R’s ksmooth function. Smoothed splines were also calculated with the ss function in the npreg package. These methods produced consistent results and produce a reasonable index of general patterns in vegetation cover as reflected in the representation of pollen in regional pollen records. n order to visualize our uncertainty and as a final check on the smoothing methods, the smoothed results are presented below with box-and-whisker plots of the binned sample results (Figure S8). This visualization reveals where outliers may have an over-sized influence on the curve (at 7500-7000 years BP and 5000-4000 years BP) and in which direction.

#### Historiographic methods

The site name (Ihò Eléérú) means “Cave of Ashes” in Yoruba language. Throughout this article the Yoruba version of the name has been anglicised into “Iho Eleru” for ease of spelling. The site was first published by the name “Iwo Eleru”, following an incorrect anglicised translation of the original Yoruba name. The Iho Eleru rock shelter was first reported as part of a large-scale survey of the hilly landscapes around Akure, conducted by Chief officer J. Akeredolu of the Department of Antiquities from Benin, Nigeria, in 1961. Following this report, in 1963 Thurstan Shaw led an exploration of the rock shelters and caves previously reported by J. Akeredolu. During the exploration of the research area, the officer of the Department of Antiquities, G. Connah, informed T. Shaw of the existence of Iho Eleru. Following the initial exploration, T. Shaw and S. Daniels organised and conducted excavations at the rock shelter between 1964 and 1965. Following two decades of research, where the material culture was studied, a final report of the excavation findings was published in 1984. Since then, several studies have been published concerning the human remains retrieved from the original Shaw and Daniels excavation, with only one article discussing the archaeological context since the original report.

#### Ecological survey methods

Today, the Iho Eleru rock shelter is situated approximately 50 km South of the modern boundary of the Guineo-Congolian rainforest phytogeographic region, with the local landscape being characterised by pioneer forest formations with a low to medium canopy cover (5-10m). Just North of this region is situated the Guinean Savanna zone, which is a large expanse of land extending West to East throughout West Africa and is characterised by forest islands and wooded savanna vegetation. The modern landscape is extensively modified by human activity, with agroforestry production focused on the cultivation of yams, bananas, cocoa, and cola nuts.

#### Geological and stratigraphy methods (Table S1)

The site is located within a lowland composed by eroded outcrops of ancient igneous deposits, with the presence of river terraces and well oxidised sandy deposits underlain by sedimentary formations of laterite. Within the region, various formations of complex metamorphic outcrops (crypto-crystalline silicates and siliceous stone formations) occur. Furthermore, Shaw and Daniels^1^ (pg. 190, Figure 3) show the presence of quartzite veins which appear approximately 5-10 km West of Iho Eleru. Furthermore, various deposits of considerably eroded quartzite and vein quartz cobbles are present within local seasonal river valleys.

Iho Eleru is a rock shelter found toward the base of an igneous inselberg, found on the western margin of the Ikere Batholith. Topographically, the main human activity area would have been behind the drip line area of the shelter, within the main upper leveled platform area (trenches D & F, XVII-XXVII). On its southern and eastern edges, steep inclined taluses slope downwards. On its western edge, a very steep incline slopes upwards toward the upper levels of the inselberg, where other sheltered areas are found. On the northern edge of the platform area, the rock shelter closes following the two overhanging igneous outcrops, forming a small tunnel (Central Tunnel) with an SW-NE orientation. The stratigraphy at Iho Eleru shows very irregular igneous beds upon which the archaeological sediments sit. The depths of the stratigraphy vary from 0 cm (subsurface outcrop) to over 2m in depth (Trench S). The sediments have post-erosional origin, where materials from the upper levels of the inselberg fall during particularly wet periods, depositing within the platform area and surrounding slopes. In turn, similar activities cause an erosion of the platform area sediments, causing the sloped areas to have inverted stratigraphies. For this reason, during our re-analysis of the chronometric dates of the site, only charcoal samples that were retrieved in the upper level area were selected. Nevertheless, the resulting ^14^C dates still show discrepancies and inverted dates. This has been interpreted as being caused by heavy erosional processes, mostly during wet season periods, which cause Iho Eleru rock shelter to become a main channelling area for water moving downstream of the upper levels of the inselberg.

Shaw and Daniels state that the stratigraphical excavation was conducted with an initial layer (spit 1) that was excavated with a total depth of 25 cm, and subsequent spits of 15 cm. However, later descriptions of the excavation show that each and every spit per square was considered to be 15 cm in depth. This contradiction might be caused by a use of a 25 cm deep spit only in specific areas of the rock shelter, however such information could not be found by the authors of this paper. For these reasons, a consistent depth of 15 cm per spit was considered and applied for the upper plateau area which was evaluated in this study, and which is consistent with the original descriptions, interpretations and excavation diagrams by Shaw and Daniels (1984).

In the platform area of the rock shelter, Shaw and Daniels describe 4 main sediment types:

These units appear to be very distinct in terms of their sedimentary properties and likely reflect changes in the local environment surrounding the rock shelter. We propose some thoughts regarding possible site formation factors operating at Iho Eleru and describe how these may relate to changes observed in our paleoclimatic modeling.

##### Gravelly soil (Table S1)

We Interpret the ‘gravelly soil’ at the base of the platform sequence to be colluvially deposited heavily weathered re-worked soils, eroded from the higher elevations of the Inselberg landform. Given that igneous bedrock and associated soils are apparently the only possible sources of colluvial sediment supply, there are limited options to explain the notable gravel component that Shaw and Daniels describe in this sedimentary horizon. We suggest that coarse components in the gravelly soil unit are likely crudely weathered elements of the igneous bedrock and/or pedogenic iron nodules re-worked from well-developed laterite soil deposits. It is also feasible that the coarse components in this unit may be derived from relict geological deposits of Quaternary, or pre-Quaternary age, situated at areas of high elevation on the inselberg, though we currently have no evidence to support this possibility. Our oldest radiocarbon age estimate for the lower spits in the platform area is 13,075–12,840 cal. BP (IW2833, F21 Spit 10). According to our paleoclimatic modeling, environmental conditions at approximately this time are predicted to be characterised by increased precipitation and humidity. Our modeled environmental expectations support our preliminary interpretations of this stratigraphic unit, whereby increased precipitation and humidity would have likely increased geochemical weathering of any exposed bedrock and increased high-intensity, precipitation may have assisted in the periodic washing of re-worked materials into the rock shelter.

##### Reddish brown soil (Table S1)

This unit is annotated by Shaw and Daniels as varying in thickness (c.1.2m–0.3m). We anticipate that where the deposit is thickest (i.e. square XX), it is likely formed of repeated depositions of re-worked soil, brought into the rock shelter by episodic pulses of in-washing. Discrete sedimentary hiatuses and stabilisation surfaces are thought to occur between pulses of in-wash. This intermittent sedimentary regime is deemed likely due to minor, *in-situ*, stratigraphic lensing that Shaw and Daniels describe, such as the layer of calcareous nodules (possibly biogenic ash) occurring at c. 0.45m below the ground surface. Well stratified horizons of rock spall clasts (Shaw and Daniels, Plt IX, pp. 182) also imply a repeated, episodic, mode of deposition. Our new radiocarbon age estimates most probably associated with this layer span c. 11,000-6,500 cal. BP. (earliest dated sample = IW2519, F21 Spit 8; youngest dated sample = IW2232, D27 Spit 6). According to our paleoclimatic models this stratigraphic unit was deposited during a modeled continuation of humid environmental conditions with high precipitation, which began approximately at c. 14,000 BP. We suggest that humid conditions are likely sustaining soil development in bedrock depressions located above the altitude of the Iho Eleru rock shelter and these soils are subject to intermittent erosion by high magnitude precipitation. The overall paucity of coarse components in this sedimentary unit might be attributed to the deposition of young, lesser developed, re-worked soil. A greater clay content in the lower portions of the ‘reddish soil’ could be attributed to phases of increased chemical weathering of the igneous bedrock, the input of a different sediment source, or sorting of the sediment’s size fraction through water agency (i.e. pooling).

##### Light red sandy layer (Table S1)

Between c. 0.5–0.2m depth, Shaw and Daniels describe a light red sandy layer. This unit is observed to have a clear and abrupt lower contact to the underlying red soil deposits (Shaw and Daniels, 1984, Plt IX) which may indicate a rapid and drastic change in sedimentary regime, perhaps in association with a small-scale erosional event. A radiocarbon sample from this unit (IW1918, D27 Spit 3), returns an age estimate of 3365–3229 cal. BP. According to our paleoclimate models, the deposition of this unit occurs during a modeled phase of increased temperature, reduced precipitation, and overall reduced precipitation seasonality. We suggest that the overall change in sedimentary regime – from re-worked soil to sandy deposits – might be linked to reduced precipitation and the cessation of soil erosion at high elevations on the inselberg landform. It is possible that the sand component of this unit derives from continued chemical weathering of the Igneous bedrock, both at high elevations and within the rock shelter itself. Aeolian deposition of fine particle material from other proximal sources, such as seasonally dried soils or alluvial environments is also possible.

##### Superficial silt (Table S1)

The sedimentary sequence at Iho Eleru is capped by c. 0.1–0.2m of mobile silts. This unit is described by Shaw and Daniels as ‘superficial ash’. This deposit is approximated to date to c. 460-314 cal. BP (IW1676, D25 Spit 1) to modern times. Two radiocarbon dates on faunal material from this unit have returned ‘modern’ ages. Recent fire making activities in the cave are likely to be the primary source of this fine-grained, mobile, ashy, material.

Further work in the field and laboratory are required to confirm the nature of the depositional sequence at Iho Eleru.

#### Material culture imaging and figure design

Photographs of the faunal remains were taken following the protocol for photography of artifacts and small object (SOAP protocol).^78^ Figures 1, 2, 3, 5 were designed and composed using Adobe © Illustrator © 2022. The maps on Figures 1A and 1B were originally designed on QGis ©. For the maps a digital elevation map of the region was merged and developed (utilising WGS 84 projection) from 1 km-resolution digital elevation models (DEMs),^79^ and then categorised based on elevation and correlated with topographic bioregions. Map b on Figure 1 was created by mapping the distribution of modern ecoregions^10^ for the landscape surrounding Iho Eleru and geolocating the respective archaeological sites. Figure 4 was designed and created using Adobe © Photoshop © 2022.

### Quantification and statistical analysis

The ages are given as mean ± ^14^C (Figure S1 and Table S4). All original raw data has been included in the Supplemental Tables with quantified data used for interpretation in this paper. Data were analyzed with RStudio (RStudio Team, 2019) running R version 3.6.2 (R Core Team, 2020).

## Data Availability

• All data necessary to interpret and replicate results are available in the main text and Supplemental Data, Supplemental Figures, and Supplemental Tables. • All original code utiilised to produce and interpret data in the paper is available within the supplemental information. • Any additional information required to reanalyze the data reported in this paper is available from the lead contact upon request.

## Supplemental information

Document S1. Figures S1–S8 and Tables S1–S5

Table S6. “Analyzed archaeobotanical samples”, related to Figure 3

Table S7. “Faunal taxonomic and taphonomic results”, related to Figure 3

Table S8. “Identified taxa of vertebrate faunal assemblage”, related to Figure 3

Data S1. Script for Paleoclimatic Reconstruction Modeling, related to STAR Methods

Data S2. Script (R Markdown) for Regional Palynological Record Modeling, related to STAR Methods
