# Supplementary material for: Human interactions with tropical environments over the last 14,000 years at Iho Eleru, Nigeria
Source: iScience. 2023 Feb 8;26(3):106153. doi: 10.1016/j.isci.2023.106153 (PMC9950523; doi:10.1016/j.isci.2023.106153)
Supplement: Document S1. Figures S1–S8 and Tables S1–S5 [file mmc1.pdf]

iScience, Volume *26*

Supplemental information

Human interactions with tropical environments over the last 14,000 years at Iho Eleru, Nigeria

Jacopo Niccolò Cerasoni, Emily Yuko Hallett, Emuobosa Akpo Orijemie, Kseniia Ashastina, Mary Lucas, Lucy Farr, Alexa Höhn, Christopher A. Kiahtipes, James Blinkhorn, Patrick Roberts, Andrea Manica, and Eleanor M.L. Scerri

Components present in this document:

- Figures S1, S2, S3, S4, S5, S6, S7, and S8
- Tables S1, S2, S3, S4, and S5

Figure S1

Calibration of new chronometric ages and original chronometric ages[1]. Related to Figure 3.

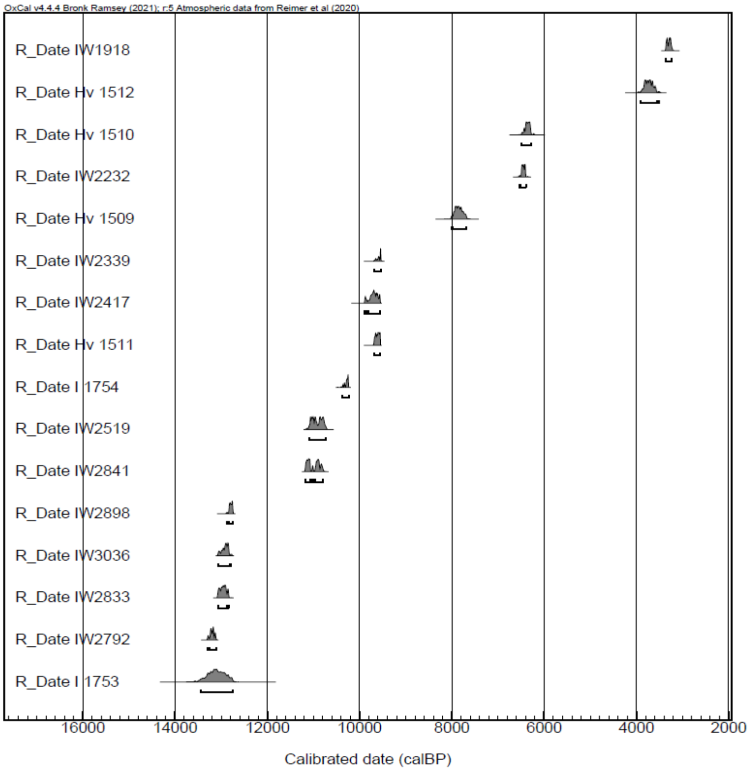

Figure S2

ZANTHOXYLUM spp., transverse (left), tangential (centre) and radial (right) section of wood charcoal fragment (IW 1918). Related to Figure 3.

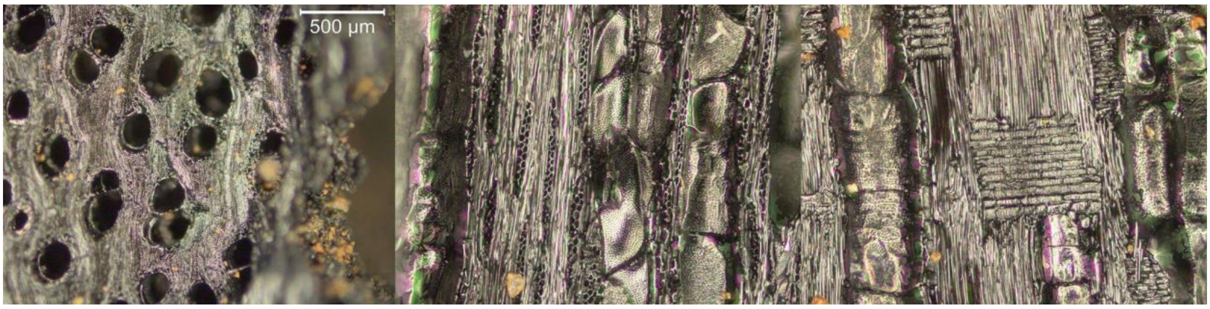

Figure S3

Selection of samples selected for isotope analysis. Related to Figure 5.

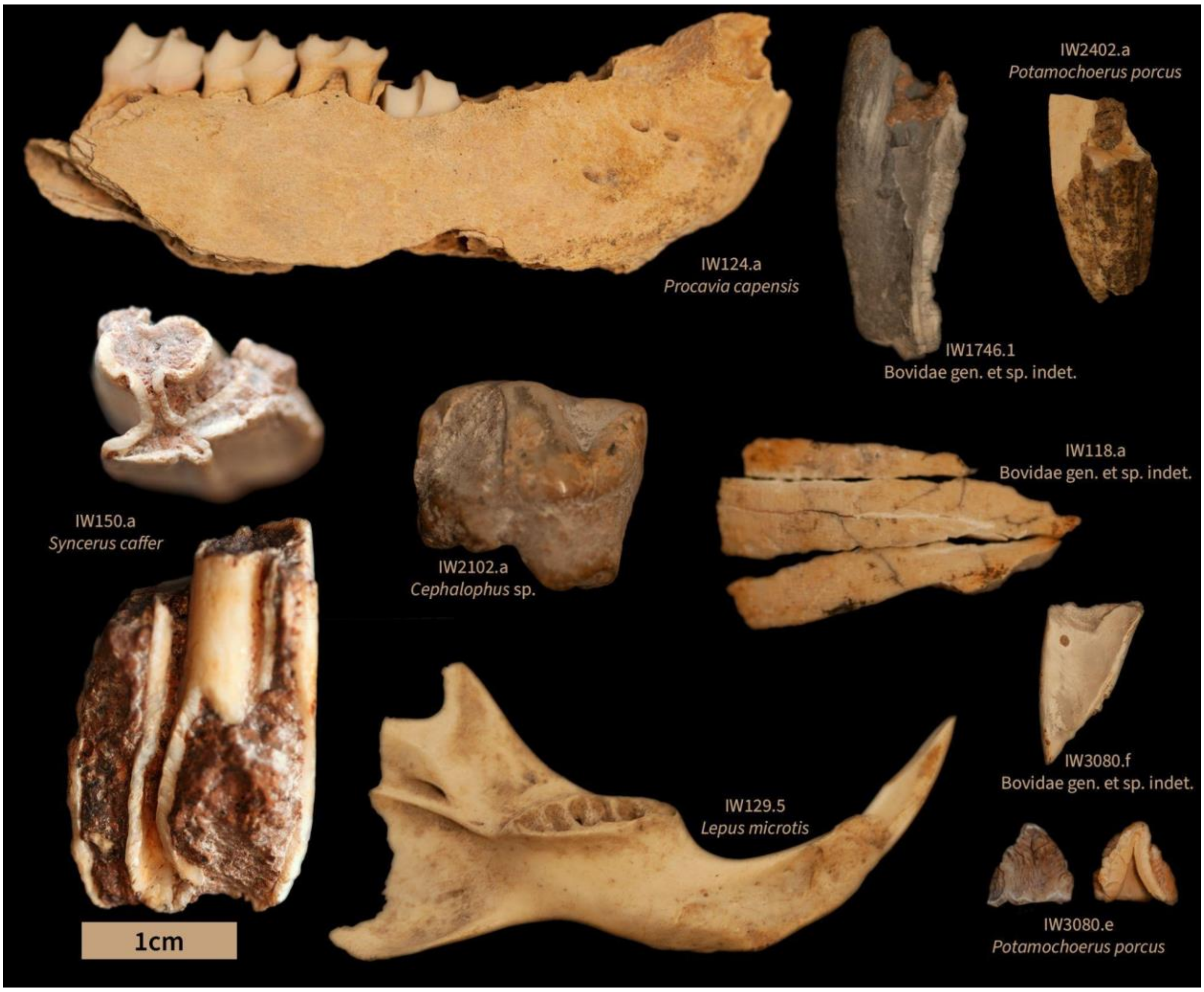

Figure S4

Modelled climate variables for each 1-thousand-year slice. Reconstructions were carried out on four separate areas. The first is at a 0.5° resolution around the site of Iho Eleru. The subsequent three are a division in longitude (0.5° each) of the total area analysed in the main study (see figure 1 and figure 6), divided as North, Central and South. Scaled climate variables (SCVV) are (see main text for legend): annual mean temperature (BIO1; orange), temperature seasonality (BIO4; green), annual precipitation (BIO12; blue), and precipitation seasonality (BIO15; purple). Related to STAR Methods.

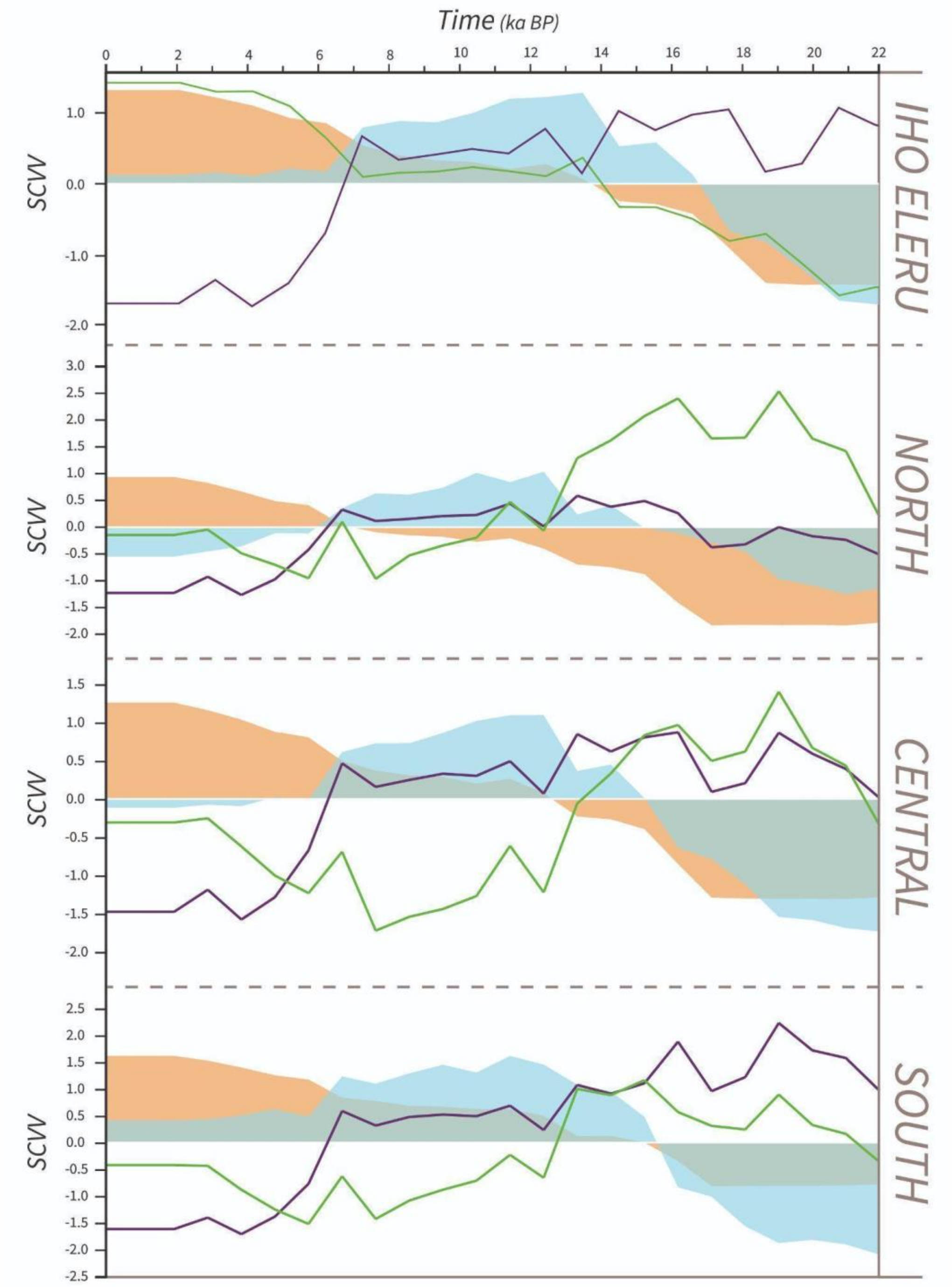

Figure S5

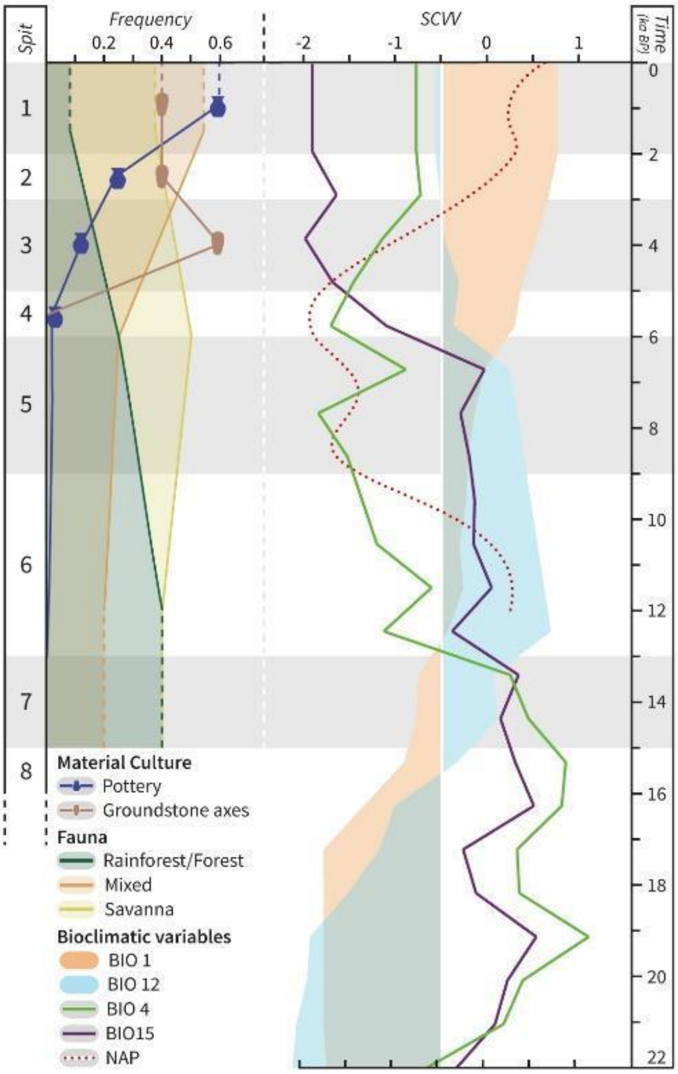

Synthesis of non-arboreal pollen (NAP), climate variable curves, material culture frequency, and faunal remains frequency during the last 22 ka. On the left are the frequency plots for the recovered material culture and identified fauna divided into environmentally categorised taxonomies. On the right are the regional non-arboreal pollen (NAP) curve and predicted variables for each 1-thousand-year slice. Scaled climate variables are: annual mean temperature (BIO1), temperature seasonality (BIO4), annual precipitation (BIO12), and precipitation seasonality (BIO15). Related to STAR Methods.

Figure S6

Map of study region showing locations of paleovegetation. Green area is tropical rain forest summarized from the Global Land Cover Characterization dataset[2]. Related to STAR Methods.

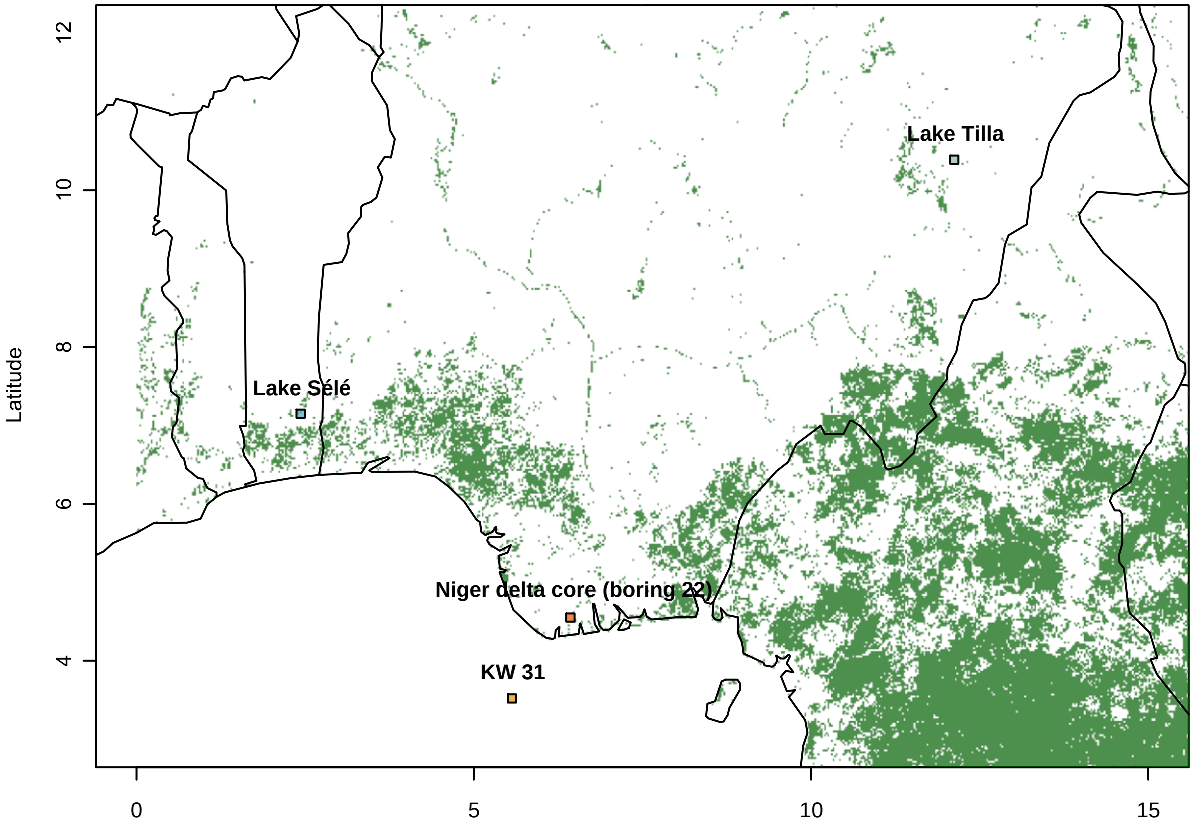

Figure S7

Plots of NAP analysis showing all data points and results of different smoothing methods. This visualization reveals where outliers may have an over-sized influence on the curve (at 7500-7000 yr BP and 5000-4000 yr BP) and in which direction. Related to STAR Methods.

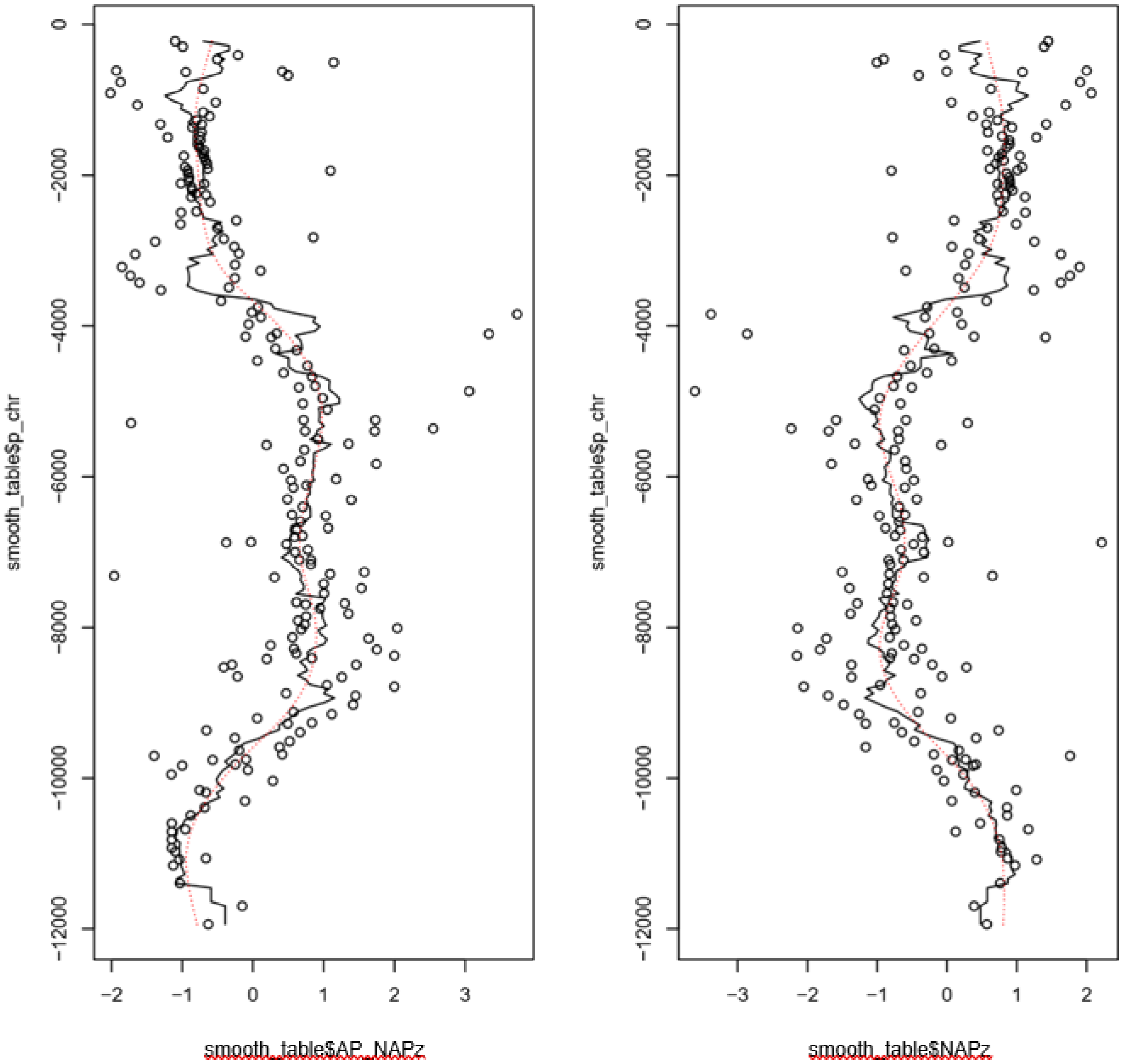

Figure S8

Box and whisker plot of data per chronological bin overlain with kernel-density smoothing results. Related to STAR Methods.

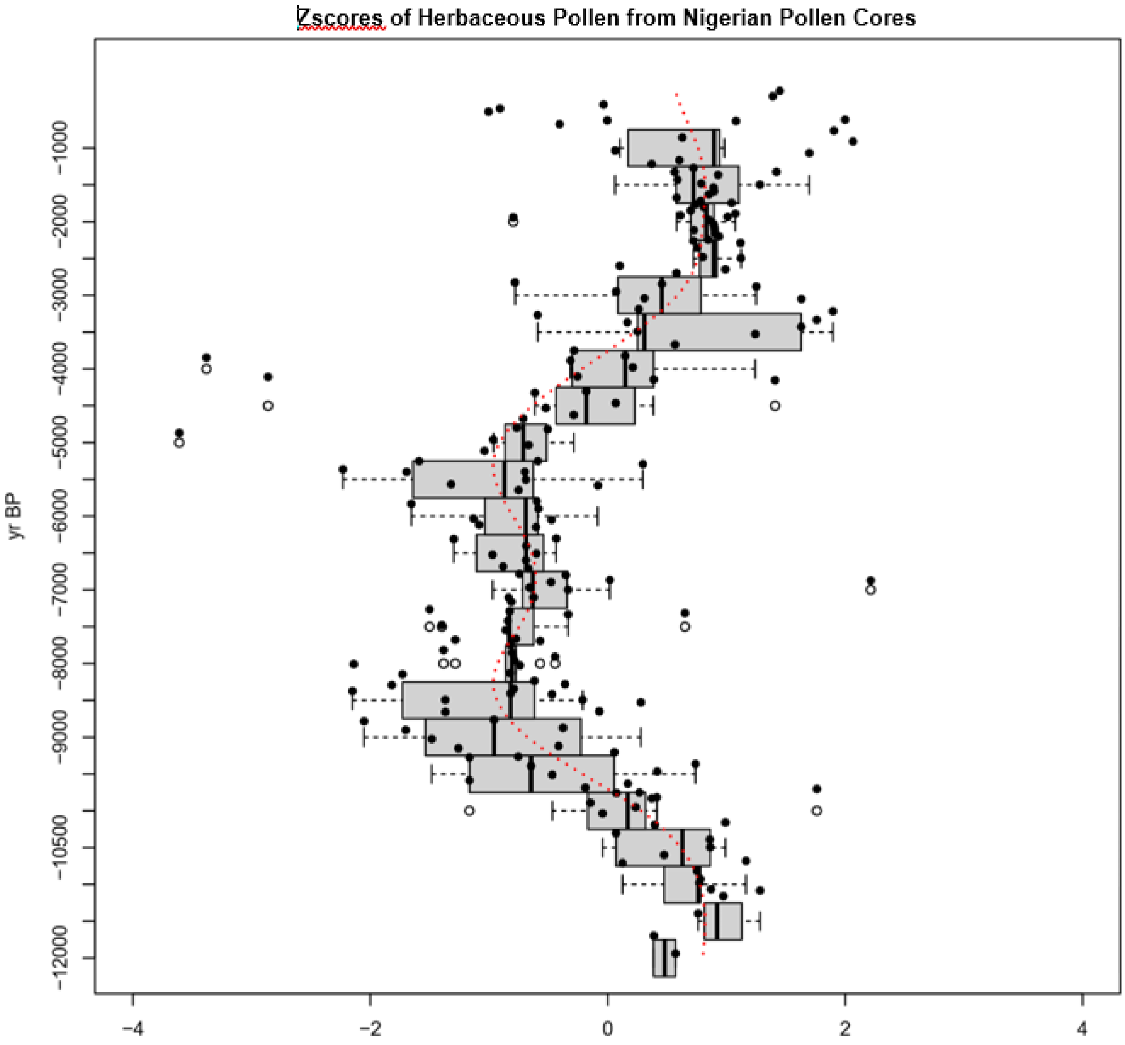

Table S1

Stratigraphic Interpretation. In the platform area of the rock shelter, Shaw and Daniels[1] describe 4 main sediment types. Related to Figure 2.

Upper section along the west face of Trench D XXV-XXVI	Lower section along the east face of Trench D XVII-XXVII
1) Superficial ash layer<br>2) Light red sandy layer<br>3) Reddish brown soil<br>4) As in 3, but rather more clayey<br>5) Gravelly soil	1) Superficial ash layer<br>2) Red sandy layer<br>3) Reddish brown soil<br>4) Gravelly soil<br>5) Browner and looser than 3, but redder and less sandy than 2 (apparent in squares XIX–XXI only)
Summarised from Shaw and Daniels[1], pp.193, figure 6.	

Table S2

List of palynological datasets used, APD (African Pollen Database[3]) information, locations, and references. Related to STAR Methods.

Name	Latitude	Longitude	Altitude (m)	Region	Reference
KW31	3.5183	5.5668	1181	Gulf of Guinea	Lézine et al., 2005[4]
Lake Sélé	5.00	2.43	NA	Southern Benin	Salzmann and Hoelzmann, 2005[5]
Lake Tilla	10.39	12.13	690	Sahel	Salzmann et al., 2002[6]
Niger delta core (boring-22)	5.50	6.43	0	Atlantic Zone	Sowunmi ,1981[7]

Table S3

Later Stone Age and early ceramic sites in West Africa. Related to Figure 1.

N. on fig. 1	Context	Country	Type	Dating	Latitude	Longitude	Reference
**1**	Iho Eleru	Nigeria	Rock shelter	14C (radiocarbon) dating	7.44138	5.12476	Shaw & Daniels, 1984[1]
**2**	Shum Laka	Cameroon	Rock shelter	14C (radiocarbon) dating	5.85861	10.07778	Lavachery, 2001[8]
**3**	Koukouan-I	Benin	Open air	Typological (lithic)	10.21710	1.04967	Petit, 2005[9]
**4**	Pendjari-II	Benin	Open air	Typological (lithic)	11.02403	0.94628	Petit, 2005[9]
**5**	Bosumpra Cave	Ghana	Rock shelter	14C (radiocarbon) dating	6.683	-0.730	Oas, D'Andrea, & Watson, 2015[10]; Watson, 2017[11]
**6**	Bingerville Highway	Ivory Coast	River terrace	14C (radiocarbon) dating	5.58500	-4.277	Chenorkian, 1983[12]
**7**	Fatandi V	Senegal	Open air	OSL (optically stimulated luminescence) dating	13.866	-12.371	Chevrier et al., 2016[13]; Lebrun et al., 2016[14]
**7**	Toumboura I	Senegal	River terrace	OSL (optically stimulated luminescence) dating	13.866	-12.371	Lebrun et al., 2016[14]
**8**	Ounjougou	Mali	Open air	OSL (optically stimulated luminescence) dating	14.532	-3.448	Huysecom et al., 2014[15]; Lebrun et al., 2016[14]; Rasse, et al., 2004[16]

Table S4

^{14}C dating results. Chronometric dating carried out at Curt Enhelhorn Zentrum Archaometrie (CEZA, Mannheim) and calibrated through OxCal v4.4.2. Related to Figure 3.

Sample Code	Context	Depth (cm)	Material	Uncalibrated ^{14}C ages (yr BP)	Calibrated ^{14}C ages (yr BP; 95% probability)
**IW1676**	D25 Spit 1	0-15	wood charcoal	334 ±16	387
**IW3036**	F19-21 Spit 2	15-30	canarium endocarp	10995 ±35	12934.5
**IW1918**	D27 Spit 3	30-45	oil palm endocarp (cf.)	3082 ±21	3297.5
**IW2339**	D23 Spit 5	60-75	unidentified botanical remain	8625 ±30	9606.5
**IW2841**	D23 Spit 6	75-90	oil palm endocarp (cf.)	9638 ±31	10987.5
**IW2232**	D27 Spit 6	75-90	canarium endocarp	5676 ±25	6467.5
**IW2792**	D23 Spit 7	90-105	canarium endocarp	11305 ±35	13207
**IW2417**	D20 Spit 8	105-120	oil palm endocarp (cf.)	8736 ±31	9722
**IW2519**	F21 Spit 8	105-120	canarium endocarp	9570 ±32	10917.5
**IW2606**	D20 Spit 9	120-135	unidentified botanical remain	2537 ±21	2621.5
**IW2833**	F21 Spit 10	135-150	canarium endocarp	11024 ±35	12958
**IW2898**	F21 Spit 11	150-165	canarium endocarp	10875 ±34	12809
**IW1050**	Ash surface	30-45 c.	faunal bone	(-)474 ±17	Modern
**IW1050**	Ash surface	30-45 c.	faunal bone	(-)617 ±18	Modern

Table S5

Isotope analysis results. Related to Figure 5.

Sample Code	Context	Taxon	$\delta^{13}C$	$\delta^{13}C$ st. dev.	$\delta^{18}O$	$\delta^{18}O$ st. dev.
**IW3080.e**	D 23 spit 6-7	*Potamochoerus porcus*	-18.6	0.1	-4.6	0.1
**IW3080F**	D 23 spit 6-7	indeterminate bovid	-18.6	0.1	-4.6	0.1
**IW118.a**	D 23 spit 6	indeterminate bovid	-13.4	0.1	-3.6	0.1
**IW1746.1**	D 22 spit 5	indeterminate bovid	-14.2	0.1	-3.6	0.0
**IW141A**	D 27 spit 2	*Cephalophus* sp.	-14.1	0.1	-3.8	0.1
**IW2102.a**	D 27 spit 4	*Cephalophus* sp.	-14.2	0.2	-8.6	0.1
**IW126A**	F 14 spit 2	*Varanus niloticus*	-14.8	0.1	-2.1	0.0
**IW124B**	F 16 spit 1A	*Lepus microtis*	-13.1	0.2	-4.2	0.1
**IW124.a**	F 16 spit 1A	*Procavia capensis*	-14.5	0.1	0.1	0.1
**IW129.5**	Spit ash surface	*Lepus microtis*	-13.6	0.1	-4.9	0.1
**IW2402.a**	G 16 spit 4	*Potamochoerus porcus*	-13.8	0.2	-3.3	0.0
**IW150.a**	Tunnel 3 spit 7	*Syncerus caffer*	2.5	0.1	1.8	0.1
**IW127.a**	Y 16 spit 1	*Hystrix cristata*	-18.4	0.1	-11.0	0.2

Supplemental References

1. Shaw, T., and Daniels, S.G. (1984). Excavations at Iwo Eleru, Ondo State, Nigeria. West African J. Archaeol 14, 1–269.

2. United States Geological Survey. USGS EROS Archive – Land Cover Products – Global Land Cover Characterization (GLCC). https://www.usgs.gov/centers/eros/science/usgs-eros-archive-land-cover-products-global-land-cover-characterization-glcc

3. Lézine, A.M., Ivory, S.J., Gosling, W.D. and Scott, L. (2021). The African Pollen Database (APD) and tracing environmental change: State of the Art. Quaternary Vegetation Dynamics–The African Pollen Database, pp.5-12.

4. Lézine, A.M., Duplessy, J.C., and Cazet, J.P. (2005). West African monsoon variability during the last deglaciation and the Holocene: Evidence from fresh water algae, pollen and isotope data from core KW31, Gulf of Guinea. Palaeogeogr. Palaeoclimatol. Palaeoecol 219, 225–237. 10.1016/j.palaeo.2004.12.027.

5. Salzmann, U., and Hoelzmann, P. (2005). The Dahomey Gap: An abrupt climatically induced rain forest fragmentation in West Africa during the late Holocene. Holocene 15, 190–199. 10.1191/0959683605hl799rp.

6. Salzmann, U., Hoelzmann, P., and Morczinek, I. (2002). Late Quaternary Climate and Vegetation of the Sudanian Zone of Northeast Nigeria. Quat. Res 58, 73–83. 10.1006/qres.2002.2356.

7. Sowunmi, M.A. (1981). Late quaternary environmental changes in Nigeria [Phytoecological groups, vegetation zones, pollen]. Pollen et spores.

8. Lavachery, P. (2001). The Holocene Archaeological Sequence of Shum Laka Rock Shelter (Grassfields, Western Cameroon). African Archaeol. Rev 18, 213–247. 10.1023/A:1013114008855.

9. Petit, L. (2005). Archaeology and History in North-Western Benin. Bar S1398.

10. Oas, S. E., D'Andrea, A. C., & Watson, D. J. (2015). 10,000 year history of plant use at Bosumpra Cave, Ghana. Veg Hist Archaeobot 24, 635–653.

11. Watson, D. J. (2017). Bosumpra revisited: 12,500 years on the Kwahu Plateau, Ghana, as viewed from 'On top of the hill.' Azania Archaeol. Res. Africa 52, 437–517. 10.1080/0067270X.2017.139

12. Chenorkian, R. (1983). Ivory Coast Prehistory: recent developments. Afr Archaeol Rev 1, 127–142. 10.1007/BF01116775

13. Chevrier, B., Rasse, M., Lespez, L., Tribolo, C., Hajdas, I., Guardiola Fígols, M. et al. (2016). West African Palaeolithic history: New archaeological and chronostratigraphic data from the Falémé valley, eastern Senegal. Quat Int, 408, 33–52. 10.1016/j.quaint.2015.11.060

14. Lebrun, B., Chantal, T., Benoît, C., Michel, R., Laurent, L., Alice, L., et al. (2016). Establishing a West African chrono-cultural framework: First luminescence dating of sedimentary formations from the Falémé Valley, Eastern Senegal. J Archaeol Sci Rep 7, 379–388. 10.1016/j.jasrep.2016.05.001

15. Huysecom, E., Loukou, Y. S. B., Mayor, A., Jeanbourquin, C., Chaix, L., Chevrier, B., et al. (2014). Vallée de la Falémé (Sénégal oriental) et Parc national des îles Eotilé (Côte d'Ivoire): la 16ème année de recherche du programme «Peuplement humain et paléoenvironnement en Afrique». Jahresbericht SLSA, 113–176.

16. Rasse, M., Soriano, S., Tribolo, C., Stokes, S., & Huysecom, E. (2004). La séquence pléistocène supérieur d'Ounjougou (Pays dogon, Mali, Afrique de l'Ouest) : évolution géomorphologique, enregistrements sédimentaires et changements culturels [The Upper Pleistocene formations of Ounjougou : geomorphological evolution, sedimen. Quaternaire 15, 329–341. 10.3406/quate.2004.1779
